# *De Novo* Sequencing and Analysis of Lemongrass Transcriptome Provide First Insights into the Essential Oil Biosynthesis of Aromatic Grasses

**DOI:** 10.3389/fpls.2016.01129

**Published:** 2016-07-28

**Authors:** Seema Meena, Sarma R. Kumar, D. K. Venkata Rao, Varun Dwivedi, H. B. Shilpashree, Shubhra Rastogi, Ajit K. Shasany, Dinesh A. Nagegowda

**Affiliations:** ^1^Molecular Plant Biology and Biotechnology Lab, Council of Scientific and Industrial Research – Central Institute of Medicinal and Aromatic Plants Research CentreBangalore, India; ^2^Biotechnology Division, Council of Scientific and Industrial Research – Central Institute of Medicinal and Aromatic PlantsLucknow, India

**Keywords:** *Cymbopogon*, aromatic grasses, transcriptome, essential oil, gene candidates, monoterpene biosynthesis

## Abstract

Aromatic grasses of the genus *Cymbopogon* (Poaceae family) represent unique group of plants that produce diverse composition of monoterpene rich essential oils, which have great value in flavor, fragrance, cosmetic, and aromatherapy industries. Despite the commercial importance of these natural aromatic oils, their biosynthesis at the molecular level remains unexplored. As the first step toward understanding the essential oil biosynthesis, we performed *de novo* transcriptome assembly and analysis of *C. flexuosus* (lemongrass) by employing Illumina sequencing. Mining of transcriptome data and subsequent phylogenetic analysis led to identification of terpene synthases, pyrophosphatases, alcohol dehydrogenases, aldo-keto reductases, carotenoid cleavage dioxygenases, alcohol acetyltransferases, and aldehyde dehydrogenases, which are potentially involved in essential oil biosynthesis. Comparative essential oil profiling and mRNA expression analysis in three *Cymbopogon* species (*C. flexuosus*, aldehyde type; *C. martinii*, alcohol type; and *C. winterianus*, intermediate type) with varying essential oil composition indicated the involvement of identified candidate genes in the formation of alcohols, aldehydes, and acetates. Molecular modeling and docking further supported the role of identified protein sequences in aroma formation in *Cymbopogon*. Also, simple sequence repeats were found in the transcriptome with many linked to terpene pathway genes including the genes potentially involved in aroma biosynthesis. This work provides the first insights into the essential oil biosynthesis of aromatic grasses, and the identified candidate genes and markers can be a great resource for biotechnological and molecular breeding approaches to modulate the essential oil composition.

## Introduction

Essential oils, also known as volatile or ethereal oils or essences, are the mixtures of highly fragrant compounds found in aromatic plants and flowers. The essential oil producing plants are distributed widely across the plant kingdom covering a large number of families including Lamiaceae (mint, basil, lavender), Rosaceae (roses), and Poaceae (aromatic grasses) (Nagegowda and Dudareva, 2006). *Cymbopogon* (aromatic grasses), the unique genus of Poaceae family known for its aromatic properties, comprises of about 180 species distributed across the world (Bertea and Maffei, 2010), of which 45 species have been reported in India (Padalia et al., 2011). These aromatic grasses are endowed with differential blend of several terpenoidal constituents and are large reserves of monoterpene rich essential oils (Devi et al., 2015). The major constituents of essential oils of these aromatic grasses comprise monoterpene alcohols geraniol (GOL) and citronellol (COL), aldehydes geranial (GAL), neral (NAL) and citronellal (CAL), and acetates such as citronellyl acetate (CA) and geranyl acetate (GA) (Khanuja et al., 2005). The essential oils of aromatic grasses have great importance in food flavors, fragrances, cosmetics, oral healthcare products, insect repellents, and aromatherapy. For instance, citral has wide industrial uses as raw material for perfumery, confectionery, Vitamin A and ionones (Moyler, 2010). Likewise, COL and its aldehyde CAL are used for manufacturing of flavor and fragrance agents. COL is also used as a raw material for the production of rose oxide (Pimentel et al., 2012). In addition, essential oils and their individual constituents from aromatic grasses possess potent pharmacological activities like cytotoxic, anti-inflammatory, antifungal, and antioxidant activities (Bayala et al., 2014).

*Cymbopogon flexuosus* (lemongrass), *C. martinii* (palmarosa), and *C. winterianus* (Java citronella), are three economically important species widely cultivated for extracting high value essential oils. These species are classified into three distinct groups based on their essential oil composition such as aldehyde type (lemongrass), alcohol type (palmarosa), and intermediate type (Java citronella) that accumulates both alcohol and aldehydes (Lavania et al., 2012). The growing global demand for *Cymbopogon* aromatic oils and their individual derivatives necessitates development of high yielding varieties that requires better understanding of genetic makeup and essential oil biosynthetic pathway in *Cymbopogon*. Breeding for essential oil improvement in *Cymbopogon* has been restricted to selection from natural populations because of problems associated with irregular flowering and seed setting. Moreover, attempts for mutation breeding have met with little success (Sharma and Ram, 2000). Hence, molecular breeding approaches using genomic resources could be promising for modulating the essential oil accumulation in *Cymbopogon*. Furthermore, understanding the biochemical and molecular mechanisms of essential oil biosynthesis could aid in metabolic engineering for enhanced essential oil production. Significant progress has been made in studying genetic analysis, chemodiversity and pharmacological effects of essential oils from *Cymbopogon* (Padalia et al., 2011; Lavania et al., 2012; Bayala et al., 2014; Rao et al., 2015). To date, reports on biochemical and molecular mechanisms of essential oil biosynthesis and molecular markers in this important genus remain very limited. As for the genomic resources, only 223 nucleotide and 180 protein sequences are reported in National Center for Biotechnology Information (NCBI) database, which provide little information on the genes responsible for the aroma formation in *Cymbopogon* species. Next generation sequencing (NGS) has emerged as a promising platform to discover novel genes, enzymes, transcription factors, and molecular markers from non-model plant species. In recent years, transcriptome approaches have been widely used for discovering and characterizing genes involved in secondary metabolic pathways (Yang et al., 2013; Rastogi et al., 2014; Niu et al., 2015) and also for identifying markers for molecular breeding (Shahin et al., 2012; Miah et al., 2013; Xu et al., 2013). Although leaf and root transcriptome analysis of citronella (*C. winterianus*) identified transcripts encoding MVA and MEP pathway genes, there was no investigation pertaining to downstream genes of essential oil biosynthetic pathway (Devi et al., 2016).

As the first step toward understanding the biosynthesis and regulation of essential oils and to generate genomic resources in aromatic grasses, here we report Illumina transcriptome sequencing, analysis and functional annotation of lemongrass (*C. flexuosus*) as a representative model for the genus *Cymbopogon*. *In silico* analysis of the transcriptome data identified several genes involved in essential oil biosynthesis, which included terpene synthases (TPS), pyrophosphatases (PPase), alcohol dehydrogenases (ADH), aldo-keto reductases (AKR), carotenoid cleavage dioxygenases (CCD), alcohol acetyltransferases (AAT) and aldehyde dehydrogenases (ALDH). Further, comparative essential oil profiling and gene expression analysis in different *Cymbopogon* sp. and homology modeling gave insights into their specific involvement in aroma biosynthesis. Also, SSR markers identified from the generated transcripotme will be useful resource for further genetic improvement by molecular breeding.

## Materials and Methods

### Plant Material, Library Preparation, and Sequencing

Mature leaves were collected from different plants of *C. flexuosus* cv. Krishna grown in field conditions (temperature 25°C ± 2°C and average humidity 60%) from CSIR-Central Institute of Medicinal and Aromatic Plants, Lucknow, India. The collected leaves were pooled and total RNA was isolated using Qiagen RNeasy mini kit (Qiagen, USA) following manufacturer’s protocol. The RNA integrity was assessed using Qubit 2.0 Fluorometer with Qubit RNA BR Assay kit (Life Technologies, USA) and on a 2100 Bioanalyzer using an Agilent RNA 6000 Pico kit (Agilent Technologies, USA). 4 μg of total RNA with an RNA Integrity Number (RIN) value of 7.0 was used for cDNA synthesis. cDNA library was prepared according to Illumina TruSeq RNA low throughput library protocol according to “TruSeq RNA Sample Preparation Guide” (Part # 15008136; Rev. A; November 2010). The library quality was assessed using 2100 Bioanalyzer using DNA 1000 kit (Agilent Technologies, USA), concentration measured using library quantification kit (Kapa Biosystems, USA) and sequencing was performed using the HiSeq2000 platform (Illumina Inc., USA) after indexing the sample and paired end library was prepared.

### Assembly and Annotation

The generated raw reads were deposited in the NCBI Short Read Archive (SRA) (SRP066939). Raw reads obtained after sequencing were filtered to obtain processed reads by removing Illumina adapter and low quality bases (*Q* < 20). *De novo* assembly was done using Velvet-1.2.09 & Oases-0.2.8 with kmer size of 31 (Zerbino and Birney, 2008; Schulz et al., 2012). Fragments Per Kilobase of transcript per Million mapped reads (FPKM) values were calculated by first aligning the trimmed reads to the assembled transcriptome using Bowtie2 program with upto 1-mismatch allowed in the seed region (length = 31bp). The assembled transcripts were annotated using BLASTX search against the NCBI Nr^1^ and UniProt^2^ database with an *E*-value cut-off of 10^-5^. GO terms were assigned to the annotated transcripts based on BLASTX hits against Universal Protein Resource (UniProt). Pathways were assigned by employing BLASTX search against Kyoto Encyclopaedia of Genes and Genomes (KEGG) using KEGG Automatic Annotation Server (KAAS)^3^. The transcription factor terms were assigned to each transcript by BLASTX search against Arabidopsis Gene Regulatory Information Server (AGRIS). The microsatellite program MISA Perl script^4^ was used for identification of SSRs. The parameters used were at least 12 repeats for mono-, 6 repeats for di-, 5 for each tri- and tetra-, 4 for each penta- and hexa- nucleotide sequences.

### Identification of Genes Related to Essential Oil Biosynthesis

Candidate transcripts were identified on the basis of their KEGG, NCBI, and UniProt annotation. The transcripts were translated using ExPASy translate tool^5^. Amino acid sequence alignment was generated using MAFFT version 7^6^ and BOXSHADE 3.21^7^. Sequence relatedness and unrooted neighbor joining phylogenetic tree with 1000 bootstrap value was generated using Molecular Evolutionary Genetics Analysis tool version 6 (MEGA 6)^8^.

### Essential Oil Extraction and GC- MS Analysis

Fresh leaf tissues (200 g) of *C. flexuosus, C. winterianus* and *C. martinii*, and inflorescence of *C. martinii* (200 g) were subjected to hydro-distillation (Li et al., 2014) using clevenger apparatus for 3h at 50°C and extracted oils were dried over Na_2_SO_4_. Gas Chromatography–Mass Spectrometry (GC–MS) analysis was performed in Agilent Technologies 7980A GC system coupled with 5977A MS detector (Agilent Technologies, USA). Essential oil was diluted 1000 times using pentane and toluene (0.0001%) was added as internal standard. One μl was injected in split mode in HP5-MS column (30 m × 250 μm with 0.25 μm film thickness). The oven temperature was adjusted to 40°C for 5 min followed by 150°C at the rate of 3°C/min, then with 5°C/min up to 200°C with a hold of 10 min and finally up to 300°C with a ramp rate of 10°C/min and a final hold for 10 min. The split ratio of 10:1 was maintained with Helium as the carrier gas at a flow rate of 1.0 ml/min. Compounds were identified using NIST/EPA/NIH MS library version 2.0g (Agilent Technologies, USA). The % composition of individual monoterpene components in different essential oils of *Cymbopogon* were determined after calibrating the peak area of internal standard toluene. The data shown is the average of three technical replicates.

### qRT- PCR Analysis

RNA isolation, cDNA synthesis and qRT-PCR analysis were performed as reported previously (Kumar et al., 2015; Singh et al., 2015). Briefly, total RNA was extracted from 100 mg tissues of *C. flexuosus* (root, leaf, and inflorescence), *C. winterianus* (leaf and inflorescence), and *C. martinii* (leaf and inflorescence) using Spectrum^TM^ Plant Total RNA Kit (Sigma–Aldrich, USA) according to the manufacturer’s instructions. In all cases, on-column DNase digestion was performed to remove trace amounts of DNA using DNase I (Sigma–Aldrich, USA). The DNA-free total RNA was quantified by UV-spectrophotometer (Kinetic Biospectrometer, Eppendorf, Germany). Two microgram of total RNA was used for first-strand cDNA synthesis with random hexamers using RevertAid H Minus Reverse Transcriptase (Thermo scientific Inc., Canada). Real-time qPCR was performed with a linear range of cDNA using Step One Real Time PCR System (Applied Biosystems, USA). For validating the stability of reference genes to be used for qPCR normalization, elongation factor 1α (*EF1α*), glyceraldehyde 3-phosphate dehydrogenase (*GAPDH*) and actin (*ACT*) were used (Supplementary Table S1). Only *EF1α* was further used for normalization because of its invariant expression under the tested conditions. qRT–PCR was performed with 5 μl of reaction volume containing 2.5 μl of 2X Maxima SYBR Green PCR master mix (Thermo Scientific, USA), 1:10 diluted cDNA and 2 μM gene-specific primers with following conditions, 94°C for 10 min for first cycle, followed by 40 cycles of 94°C for 15 s. 60°C for 15 s. Fold change differences in gene expression were analyzed using the comparative cycle threshold (Ct) method (Applied Biosystems, USA). All experiments were repeated using three technical replicates and data were analyzed statistically (±SD).

### Homology Modeling and Docking

Homology models were built by comparative protein modeling with the help of MODELER 9.15 (Sali and Blundell, 1993), and validated using Ramachandran plot. The 3D coordinates of template structures were obtained from Protein Data Bank (PDB)^9^ by using BLASTP. The homology models of CfADH1 and CfADH2a were built using the X-ray structures of *Populus tremuloides* synapyl alcohol dehydrogenase (PDB ID: 1YQD), which shared 74% sequence identity. The 3D structure for AKR2b was generated using its suitable experimental structure homolog, perakine reductase from *Rauvolfia serpentina* (PDB ID: 3V0T), which shared 55% sequence identity. Hydroxycinnamoyl transferase (PDB ID: 4G0B; 30% sequence identity) structure from *Coffea canephora* and ALDH X-ray structure from *Bos taurus* (PDB ID: 1AG8; 62% sequence identity) were used to develop the homology models for CfAAT3 and CfALDH3, respectively. The models were visualized by Python Molecular Viewer (PMV) (Sanner, 1999). Homology models were modified by adding Kollman charges and polar hydrogen using ADT command. Docking studies were done using Autodock suite (Goodsell et al., 1996). The 3D substrate structures were created using Java molecular editor, and the structure topologies and energy minimization were carried out with GROMOS87 force fields with PRODRG suite (Schüttelkopf and van Aalten, 2004). Possible binding sites of substrates on candidate proteins were obtained by defining pregrids using autogrid command, and docking was done with Lamarckian genetic algorithm (LGA). The 50 LGA runs were performed in a defined grid map. Low RMSD and lowest ΔG of binding between receptor and the substrate compound was the criteria used for defining the possible binding site. The substrate bound complexes were visualized by PMV software.

## Results and Discussion

### *De Novo* Assembly and Functional Annotation

Aromatic grasses have been widely studied in terms of their essential oil composition but there are limited studies on its essential oil biosynthesis, which could be due to non-availability of genomic resources. Here, we have generated *C. flexuosus* leaf transcriptomic data using Illumina HiSeq2000 platform that has been used for sequencing many economically important monocots (Shahin et al., 2012; Davey et al., 2013). The transcript assembly details are provided in **Table 1**. Majority of the transcripts were between 150 and 1000 bases (75,261 transcripts) followed by 1000–2000 bases (14,565 transcripts) (Supplementary Figure S1A). The average length (635 bases), GC content (49.89%), and N50 values for the assembled transcripts were within the range of other monocot transcriptomes from *Zea mays* (Hansey et al., 2012), *Lilium* genus (Shahin et al., 2012; Du et al., 2015), and *Musa balbisiana* (Davey et al., 2013), which were assembled using the same sequencing platform. Of the total transcripts, 92,139 (99%) with FPKM of >1 (Supplementary Figure S1B) were subjected to BLASTX against different databases and the annotation summary is provided in Supplementary Table S2. The results showed that 82.80% (76,293) transcripts had at least one significant hit against NCBI non-redundant (Nr) protein database, with ∼ 98% having similarity of >60% at protein level, indicating high protein conservation (Supplementary Figures S1C,D).

**Table 1:** RNA sequencing summary of *C. flexuosus* leaf transcriptome.

Summary of RNA-Seq	*C. flexuosus*
Total Number of HQ Reads	26936556 (26.93 Mb)
Number of paired-end reads after trimming	23793466 (23.79 Mb)
Mean read quality (Phred score)	33.85
Number of bases (MB)	2720.59
Number of bases (GB) after trimming	1.89
Mean read length (bases)	101
kmer size	31
Number of assembled transcripts	107,363
Number of transcripts with length ≥ 150 bases	92,937
Maximum transcript length (bases)	47,050
Average transcript length (bases)	635
N50 value	968
Mean GC % of transcripts	49.89
Number of transcripts with FPKM ≥1.0	92,139

Organism distribution in NCBI annotations showed presence of 906 organisms that contained homologous genes for *C. flexuosus* transcripts with top 5 organisms belonging to Poaceae family. *Oryza sativa* ssp. japonica showed highest coverage with 39.6% (30,235) followed by *Sorghum bicolor* and *Z. mays* with 23.5% (17,909) and 14.36% (10,962), respectively (Supplementary Figure S2). In total, 69,984 transcripts represented 5,281 GO terms distributed among three main ontologies comprising molecular function (MF), cellular components (CC), and biological processes (BP) (Supplementary Figure S3). Various GO assignments of classified unigenes revealed the diversity of transcripts represented in lemongrass transcriptome. Since, trancription factors (TFs) play key role in regulating gene expression and metabolite accumulation in different metabolic pathways, a BLASTX search against AGRIS was performed that resulted in 5,867 transcripts (6.37%) with “transcription factor activity” belonging to 44 known TF families (Supplementary Figure S4). The top 5 hits of TFs were represented by Trihelix, C2H2, C3H, bHLH, and WRKY class. Further analysis and characterization of TFs from the transcriptome data could give insights into their role in regulating monoterpene biosynthesis in *Cymbopogon*. Overall, 76,409 (82.92%) transcripts significantly corresponded to known or unknown proteins present in public databases. A significant number of 15,730 (17.07%) transcripts remained unannotated, which may belong to untranslated regions, non-coding RNAs, intergenic spacers (Liu et al., 2012), or may be unique to *C. flexuosus* and can be a great resource for the discovery of novel genes.

### Secondary Metabolic Pathways Identified in *C. flexuosus* Leaf Transcriptome

**FIGURE 1:**
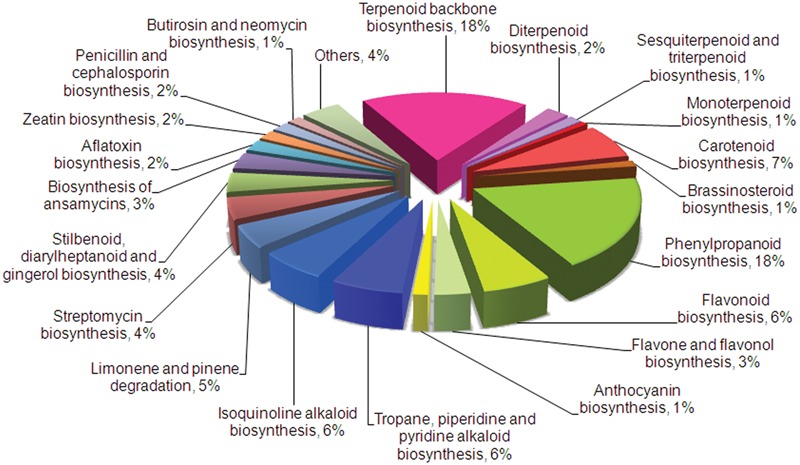
**KEGG classifications of genes involved in secondary metabolism in *Cymbopogon flexuosus* leaf transcriptome.** Relative percentage of transcripts involved in various secondary metabolic pathways is shown.

In order to find transcripts related to secondary metabolic pathways, assembled transcripts were annotated against KEGG, NCBI, and UniProt databases. About 26.21% (24,147) transcripts were assigned to 280 KEGG pathways among different categories that included metabolism, cellular processes, genetic information processing, environmental information processing, and others, with “metabolism” having the highest share of 36% (8,719 transcripts) (Supplementary Figure S5). Within the “metabolism” category, 38 metabolic pathways related to secondary metabolism (484 transcripts) were identified with terpenoid biosynthesis representing the largest group with 151 transcripts (**Figure 1**). This is in accordance with higher amounts of terpenes present in *C. flexuosus* essential oil. KEGG analyses of transcriptomes from terpene rich *Ocimum basilicum* and phenylpropanoid rich *Ocimum sanctum* also showed higher representation of respective pathway related transcripts (Rastogi et al., 2014), indicating that the distribution of transcripts involved in secondary metabolism correlates with the essential oil composition. Also, significant number of transcripts involved in the biosynthesis of secondary metabolites like phenylpropanoids (18%), alkaloids (12%), and flavonoids (10%) were represented. The total number of transcripts encoding enzymes involved in major metabolic pathways obtained from KEGG, NCBI, and UniProt annotations are represented in Supplementary Tables S3 and S4.

### Identification of Transcripts Related to Terpenoid Metabolism

**FIGURE 2:**
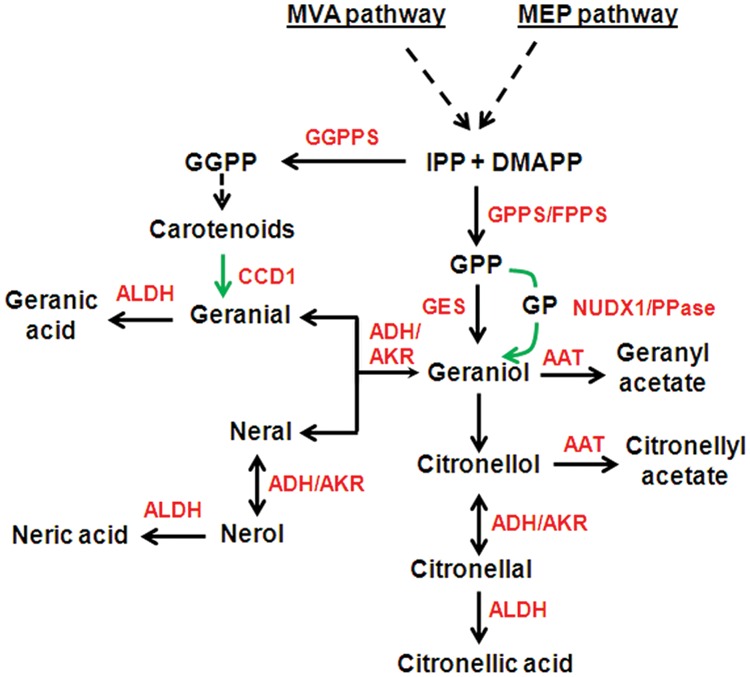
**Biosynthetic pathway of essential oil biosynthesis in *C. flexuosus*.** Broken arrows indicate multiple steps. Green arrows indicate alternate route of biosynthesis or already characterized steps in other plants. Double headed arrow indicates bidirectional reaction. Sequences analyzed in this study is shown in red color. AAT, alcohol acetyltransferase; ADH, alcohol dehydrogenase; AKR, aldo-keto reductase; ALDH, aldehyde dehydrogenase; CCD, carotenoid cleavage dioxygenase, DMAPP, dimethylallyl diphosphate; FPPS, Farnesyl diphosphate synthase; GES, geraniol synthase; GPP, Geranyl diphosphate; GP, Geranyl monophosphate; GPPS, GPP synthase; GGPP, Geranylgeranyl diphosphate; GGPPS, GGPP synthase; IPP, isopentenyl diphosphate; NUDX, nudix hydroxylase; PPase, pyrophosphatase.

The essential oil of *Cymbopogon* comprises mainly of monoterpene alcohols (GOL, COL), aldehydes (GAL, NAL, CAL), and acetates (GA, CA). The schematic representation of the steps involved in biosynthesis of these compounds is shown in **Figure 2**. Terpenoids are derived from two five-carbon precursors, isopentenyl diphosphate (IPP) and its isomer, dimethylallyl diphosphate (DMAPP) *via* the cytosolic mevalonic acid (MVA) pathway and plastidial methylerythritol phosphate (MEP) pathway (Nagegowda, 2010). In *C. flexuosus* transcriptome, 56 and 77 transcripts were annotated for 7 genes of MEP pathway and 6 genes of MVA pathway, respectively (Supplementary Table S3). IPP and DMAPP are further converted to geranyl diphosphate (GPP), farnesyl diphosphate (FPP), and geranylgeranyl diphosphate (GGPP), by GPP synthase (GPPS), FPP synthase (FPPS), and GGPP synthase (GGPPS), respectively (Nagegowda, 2010). In this analysis, we identified 8 transcripts for *GPPS*, 9 transcripts each for *FPPS* and *GGPPS* (Supplementary Table S3). In general, plants produce monoterpenes from GPP, which is formed by GPPS. Both homomeric and heteromeric forms of GPPS have been reported in plants (Rai et al., 2013), however, mostly heteromeric GPPS have been shown to be involved in monoterpene bisoynthesis (Wang and Dixon, 2009). Although *C. flexuosus* contained transcripts for both homomeric and heteromeric GPPS (Supplementary Table S3), only heteromeric GPPS may be involved in monoterpene biosynthesis similar to dicots (Rai et al., 2013).

Geranyl diphosphate is further converted to GOL either by geraniol synthase (GES) in plastids (Simkin et al., 2013) or through recently proposed NUDX1 of Nudix hydrolase superfamily in the cytosol (Magnard et al., 2015) or by yet to be identified PPase (Nah et al., 2001) (**Figure 2**). We identified 16, 26, and 17 transcripts for TPS, NUDX, and PPase, respectively, which could play a role in GOL biosynthesis (Supplementary Table S3). It is also proposed that GOL could be formed from GAL through the action of ADH/AKR (Iijima et al., 2014; Sato-Masumoto and Ito, 2014). Genes involved in downstream conversion of GOL into its aldehyde, acetate or acid derivatives involve ADH/AKR, AAT, and ALDH at their respective steps of synthesis (**Figure 2**). Our search resulted in 92 ADHs, 38 AKRs, 35 AATs, and 88 ALDHs (Supplementary Table S3). Aldehydes (GAL and NAL) could also be synthesized by a novel route utilizing carotenoids as substrate through the action of CCDs as reported in rice and tomato (Ilg et al., 2009, 2014). *C. flexuosus* transcriptome contained 11 transcripts annotated as CCDs (Supplementary Table S3).

### Mining of Genes Related to Essential Oil Biosynthesis in *C. flexuosus*

For mining of candidate genes, only those transcripts encoding full length proteins were considered for further analyses. Search for candidates involved in GOL formation yielded 1, 6, and 8 genes encoding TPS (Supplementary Figure S6), PPase, and NUDX, respectively. BLAST analysis of *C. flexuosus* putative NUDX candidates showed no significant homology to the recently characterized NUDX from *Rosa hybrida* (RhNUDX1) (Magnard et al., 2015). Also, when RhNUDX1 was used to search homologous candidates against NCBI monocot and other databases including Oryzabase^10^, Rice Genome Annotation Project^11^, Phytozome v10.3^12^ and PlantGDB^13^, it did not yield any homologous NUDX candidates except in *Zostera marina* (seagrass) (52% identity) of Zosteraceae family. The obtained results could possibly be due to the reason that *Z. marina* is more close to dicots, further suggesting that divergence of monocots and dicots occurred after *Z. marina* got established as a marine plant (Kong et al., 2014). Since there were no homologs for RhNUDX1 in *C. flexuosus*, only TPS and PPase were considered for further analyses. Although GES enzymes involved in the formation of GOL have been characterized in dicots (Iijima et al., 2004; Dong et al., 2013; Simkin et al., 2013), they are yet to be identified in monocots. The identified CfTPS1 in this study had the closest similarity (63% identity) with putative LIS/NES from rice. Among the characterized GES from other plants, *Vitis vinifera* GES exhibited a low similarity (36% identity) to CfTPS1 (Supplementary Figure S6; Supplementary Table S5). As for the PPase, it has been reported that in rice seedlings FPPase and GGPPase activities are involved in formation of farnesol and geranylgeraniol from FPP and GGPP, respectively (Nah et al., 2001). A similar phosphatase activity could be possibly involved in GOL formation from GPP in monocots. However, the molecular evidence for such activity still needs to be determined.

**FIGURE 3:**
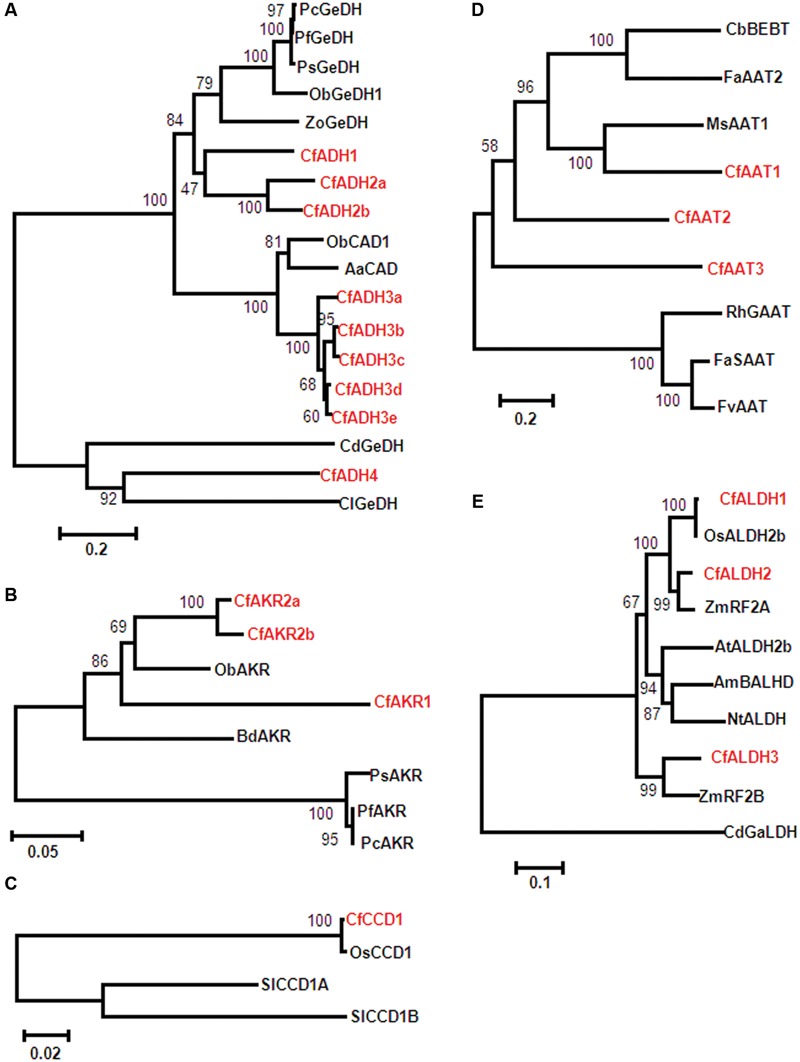
**Phylogenetic relationship of *C. flexuosus* candidate sequences.** The evolutionary relationship was analyzed by unrooted Neighbor–Joining (NJ) using MEGA6 (Tamura et al., 2013). The branch length of the line indicates evolutionary distance and numbers represent confidence of the phylogenetic tree calculated by bootstrap analysis from 1000 replicates. Phylogenetic analysis of ADH **(A)**, AKR **(B)**, CCD **(C)**, AAT **(D)**, and ALDH **(E)** candidates. The abbreviation and accession numbers are: **(A)** CAD, cinnamayl alcohol dehydrogenase; GeDH, geraniol dehydrogenase; Aa, *Artemisia annua* (ACB54931); Cd, *Castellaniella defragrans* (WP_043683915); Cl, *Carpoglyphus lactis* (B2N193); Cf, *C. flexuosus*; Ob, *Ocimum basilicum* (ObCAD1_Q2KNL5, ObGeDH1_AAX831C7); Pc, *Perilla citriodora* (AFY63473); Pf, *Perilla frutescens* (AFY63472); Ps, *Perilla setoyensis* (AFY63474); Zo, *Zingiber officinale* (BAR42579). **(B)** Bd, *Brachypodium distachyon* (XP_003575318); Cf, *C. flexuosus;* Ob, *Oryza brachyantha* (XP_006652179); Pc, *P. citriodora* (AFV99149); Pf, *P. frutescens* (AFV99148); Ps, *P. setoyensis* (AFV99150). **(C)** Cf, *C. flexuosus;* Os, *Oryza sativa* (AK066766); Sl, *Solanum lycopersicum* (SlCCD1A_AAT68187 and SlCCD1B_AAT68188). **(D)** Ms, *Musa sapientum* (CAC09063); CbBEBT, *Clarkia breweri* benzyl alcohol O-benzoyltransferase (AAN09796), FaSAAT- *Faragaria ananassa* alcohol acyltransferase (AAG13130); FvAAT2, *Fragaria vesca* alcohol acyltransferase (AAN07090); RhGAAT, *Rosa hybrida* acetyl CoA geraniol/citronellol acetyltransferase (AAW31948). **(E)** AmBALHD, *Antirrhinum majus* benzaldehyde dehydrogenase (ACM89738); At, *Arabidopsis thaliana* (Q8S528); CdGaLDH, *C. defragrans* geranial dehydrogenase (CCF55023); Cf, *C. flexuosus;* Nt, *Nicotiana tabacum* (CAA71003), Os, *O. sativa* (AAF73828); ZmRF, *Zea mays* restoration factor (ZmRF2A_AAC49371, ZmRF2B_AAL99613).

Members of MDR superfamily (medium chain dehydro genases/reductases) such as cinnamyl alcohol dehydrogenases (CAD) and ADH families have been reported in citral formation. In this study, among 46 transcripts encoding ADH proteins, 9 sequences (denoted as CfADH1, CfADH2a-b, CfADH3a-e, and CfADH4) showing high homology to already characterized geraniol dehydrogenase (GeDH) and CAD (with GeDH activity) were considered for their relatedness through phylogeny (**Figure 3A** and Supplementary Table S5). CfADH1, CfADH2a, and CfADH2b were clustered in the CAD class-II having specific GeDH activity catalyzing conversion of GOL/nerol (NOL) to GAL/NAL (**Figure 3A** and Supplementary Table S5). CfADH1 exhibited highest amino acid identity (59%) with *Zingiber officinale* (ZoGeDH1) that catalyzes bidirectional inter-conversion of GOL to GAL (Iijima et al., 2014). CfADH2a and CfADH2b exhibited highest identity of 65 and 62% identity, respectively, to ZoGeDH1 (Iijima et al., 2014) (**Figure 3A** and Supplementary Table S5). CfADH3a-e formed a different clade with multifunctional CAD class-I members, sharing 77% identity with *O. basilicum* ObCAD1 (Iijima et al., 2006) and 74% identity with *Artemisia annua* AaCAD (Li et al., 2012) that exhibited catalytic ability toward different aliphatic and aromatic aldehydes/alcohols including GAL/GOL. CfADH4 formed a separate clade with members of benzyl alcohol denydrogenase (benzyl/aryl ADHs) family exhibiting GeDH activity. CfADH4 shared 35 and 31% identity to GeDH from astigmatid mite *Carpoglyphus lactis* (Noge et al., 2008) and *Castellaniella defragrans* (Lüddeke et al., 2012), respectively. Search for AKRs (members of ADH family) yielded 3 candidates showing maximum homology to the characterized AKRs from *Perilla* (Sato-Masumoto and Ito, 2014) involving GOL to GAL conversion with CfAKR1 sharing 62% identity; CfAKR2a and CfAKR2b having 68% identity (**Figure 3B** and Supplementary Table S5). From 7 transcripts encoding CCD proteins, one sequence named as CfCCD1 exhibited very high homology with rice (99%) and tomato (74–78%) CCDs that have been recently characterized to produce GAL and NAL by breaking C_7_–C_8_/C_7_ = C_8_ bonds (Ilg et al., 2009, 2014) (**Figure 3C**, Supplementary Figure S12; Supplementary Table S5).

With respect to AAT enzymes involved in GA/CA formation, 6 AATs have been identified from plants including banana AAT1(Beekwilder et al., 2004), *Clarkia breweri* benzyl alcohol *O*-benzoyltransferase (Dudareva et al., 1998), *Fragaria ananassa* AAT (Cumplido-Laso et al., 2012), *F. chiloenisis* AAT (González et al., 2009), and *R. hybrida* acetyl CoA geraniol/citronellol acetyltransferase (RhGAAT1) (Shalit et al., 2003). Out of 8 AATs mined, CfAAT1, CfAAT2, and CfAAT3 showing 28, 40, and 23% identity, respectively, to banana AAT1, and RhGAAT1 were considered for further analyses (**Figure 3D** and Supplementary Table S5).

Geranic acid (GAc), an oxygenated monoterpene present in trace amounts in *Cymbopogon* sp. has anticancer/tyrosinase inhibitor and antifungal activity (Yang et al., 2011). GAc has been reported to be formed from GAL by ALDH enzyme. ALDH acting on GAL has so far only been reported from *C. defragrans* that catalyzes the NAD^+^ dependent oxidation of GAL to GAc (Lüddeke et al., 2012). Of 18 candidates encoding ALDH proteins, 3 CfALDH sequences were closely related to previously characterized ALDH from other plants (**Figure 3E** and Supplementary Table S5). While CfALDH1 shared highest homology with uncharacterized OsALDH2b (99%) from rice, CfALDH2 and CfALDH3 had close relationship with maize fertility-restorer (RF) genes ZmRF2B (84%) and ZmRF2A (95%), respectively (Cui et al., 1996; Liu et al., 2001) (**Figure 3E** and Supplementary Table S5). All CfALDH proteins showed ∼37% identity with characterized *C. defragrans* geranial dehydrogenase (GALDH) (**Figure 3E** and Supplementary Table S5).

### Comparative Essential Oil Profiling and Gene Expression Analysis

**FIGURE 4:**
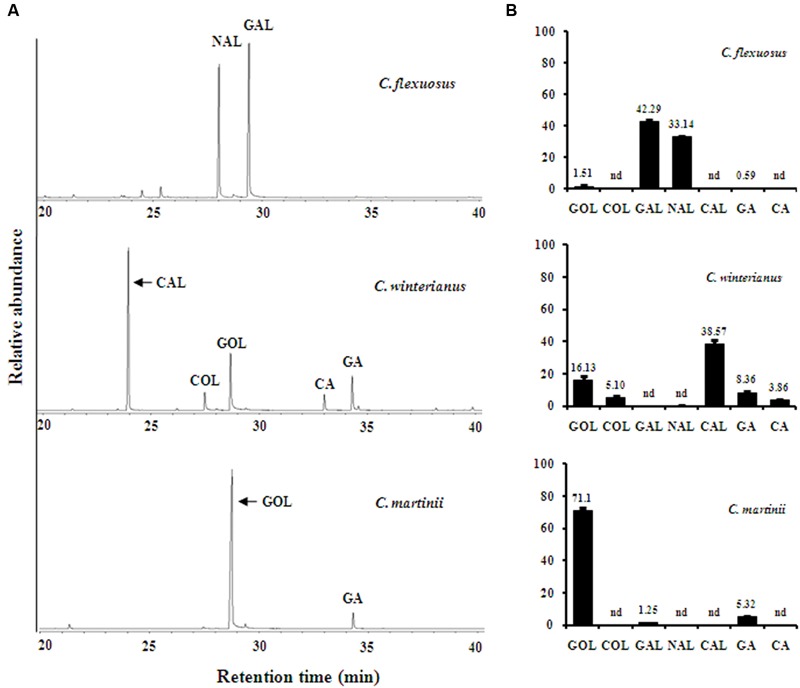
**Gas Chromatography–Mass Spectrometry (GC–MS) profile representing relative abundance of essential oil components in *Cymbopogon*.** Essential oils were hydrodistilled from 200 g fresh leaf tissue of *C. flexuosus, C. winterianus*, and *C. martinii*. One micro litre of diluted oil (1:1000 in pentane) was analyzed by GC–MS using HP5-MS column **(A)**. The compounds were identified using NIST/EPA/NIH mass spectral library version 2.0 g and relative abundance of individual components was determined from pooled leaf samples with three technical replicates **(B)**. Abbreviations: CA, citronellyl acetate; CAL, citronellal; COL, citronellol; GA, geranyl acetate; GAL, geranial; GOL, geraniol; NAL, neral; nd, not detected.

The chemical diversity in essential oil imparts different fragrances to aromatic grasses, which could be due to the differential expression of genes involved in conversion of basic substrate into their derivatives. Also, it has been reported in many plant species that the level of terpenoid volatiles correlates with the expression of corresponding genes (Nagegowda, 2010). Hence, comparative gene expression and metabolite analyses could further facilitate narrowing down the possible gene candidates involved in essential oil formation. Since strong homology of gene sequences exists among the closely related species within the same genus, gene specific primers, designed based on lemongrass transcriptome, were used for determining the expression levels in all three *Cymbopogon* species that accumulate varying composition of monoterpenes (Lavania et al., 2012). First, leaf essential oils of *C. flexuosus, C. winterianus, C. martinii*, and inflorescence of *C. martinii* were analyzed (**Figure 4A** and Supplementary Figure S7). *C. flexuosus* (group I - aldehyde type) was rich in GAL (42%) and NAL (33%) which are together called as citral with relatively trace amounts of GOL and GA (**Figure 4B**). Essential oil of *C. winterinus* (group II- intermediate type) was well represented by different levels of various monoterpene derivatives with CAL (39%) representing the major constituent followed by 21% of alcohols (GOL and COL) and 11% of acetates (GA and CA) (**Figure 4B**). Essential oils from leaf and inflorescence of *C. martinii* were dominated by GOL (71 and 57%) and GA (5 and 15%) (**Figure 4B** and Supplementary Figure S7).

**FIGURE 5:**
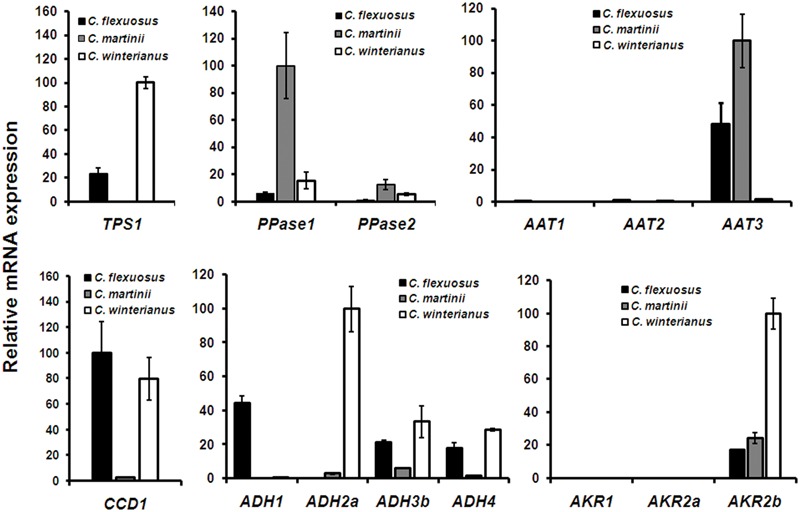
**Comparative expression of candidate genes in *Cymbopogon*.** Real-time qPCR analysis of *TPS, PPase, CCD, ADH, AKR*, and *AAT* candidates in *C. flexuosus, C. martinii*, and *C. winterianus.* While the expression of all candidates except *AAT* was compared in leaf tissue, the expression of *AAT* was analyzed in inflorescence. In each graph, the species/gene having the least *Ct* was set to 100 to determine the relative abundance of transcripts in other species. *CfEF1*α was used as endogenous control for normalization. Each data point represents the mean ± standard error (SE).

Next, the expression of identified candidate genes was compared in *Cymbopogon* that were used for essential oil analysis. Before proceeding for qPCR analysis of candidate genes, endogenous reference genes were selected and validated for transcript normalization. Among the selected reference genes, *EF1α* exhibited highest stability across different *Cymbopogon* species and in different tissues (Supplementary Figure S8A). Hence, *EF1α* was used for normalization in all subsequent qPCR analysis. For analysis, the species/gene having the least *Ct* was set to 100% to determine the abundance of transcripts relative to other species/genes. The expression of *TPS1* was highest in *C. winterianus* followed by *C. flexuosus* (24%) and negligible in *C. martinii* (**Figure 5**). Tissue specific expression of *CfTPS1* in *C. flexuosus* indicated minimal expression in root as compared to leaf (Supplementary Figure S8C). *PPase1* exhibited highest expression in *C. martinii* followed by *C. winterianus* (15%) with least in *C. flexuosus* (5%). Although *PPase2* followed similar trend as that of *PPase1*, the expression of *PPase1* was ∼ three to eightfold higher compared to *PPase2* in all three species. The GOL content in *C. flexuosus* has been reported to be 4 and 0.4% in leaf and root, respectively (Rao et al., 2015). Hence, to determine the involvement of *PPases*, tissue specific expression was studied, which showed a differential trend of *PPase* expression. While *PPase1* showed fivefold higher expression in leaf compared to root, *PPase2* exhibited sevenfold higher abundance in root (**Figure 5** and Supplementary Figure S8C). Based on the GOL content, comparative and tissue specific expression, we implicate the involvement of *PPase1* in GOL formation in aromatic grasses, similar to involvement of FPPase and GGPPase in farnesol and geranylgeraniol formation in rice (Nah et al., 2001).

**FIGURE 6:**
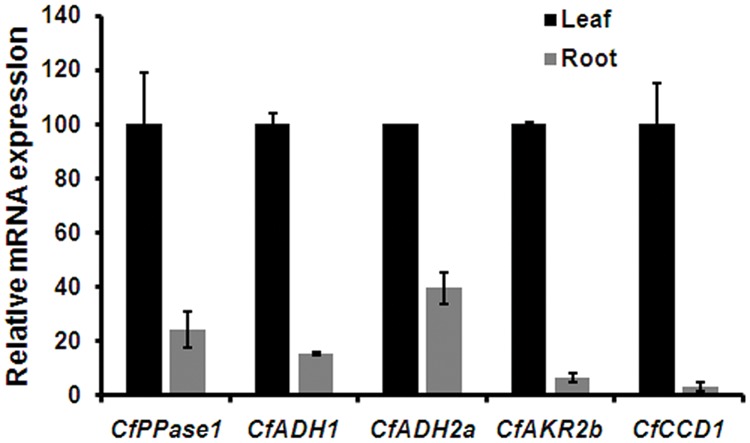
**Tissue specific expression of candidate genes in *C. flexuosus*.** Real-time qPCR analysis of candidate genes in leaf and root of *C. flexuosus*. The expression in leaf was set to 100 to determine the relative abundances of mRNA in root. *CfEF*1*α* was used as endogenous control for normalization. Each data point represents the mean ± standard error (SE).

As aldehydes (GAL, NAL, and CAL) are reported to be formed *via* different routes involving ADH/AKR (Iijima et al., 2006; Sato-Masumoto and Ito, 2014) and CCD (Ilg et al., 2009, 2014), the expression of transcripts encoding the respective enzymes were analyzed. Of 9 *ADH* candidates used for phylogeny (**Figure 3A**), *ADH1, ADH2a, ADH3b*, and *ADH4* having highest FPKM were used for expression analysis. In addition, all 3 *AKR* candidates were used for expression analyses. All 4 *ADH* candidates showed differential expression with varying levels among the three species analyzed (**Figure 5**). While expression of *ADH2a, ADH3b, ADH4*, and *AKR2b* was highest in *C. winterianus, ADH1* exhibited highest expression in *C*. *flexuosus*. *AKR1* and *AKR2a* showed basal level of expression in all species as compared to *AKR2b* (**Figure 5**). The expression of all four *ADH* candidates was least in *C. martinii* and *AKR2b* was least in *C. flexuosus* (**Figure 5**). In *C. flexuosus*, the expression of *ADH1, ADH2a*, and *AKR2b*, and *ADH3b* and *ADH4* was higher in leaf and root, respectively (**Figure 6** and Supplementary Figure S8C). The species and tissue specific expression of *ADH1*, and *ADH2a* and *AKR2b* was in agreement with citral and CAL content, respectively (Rao et al., 2015) (**Figures 4B** and **5**). A similar trend in *ZoGeDH* expression and GAL content was reported in ginger tissues (Iijima et al., 2014). The higher expression of *ADH3b* and *ADH4* in roots compared to leaf implied that they may not be involved in monoterpene aldehyde formation (Supplementary Figure S8C). The transcript encoding CCD1 exhibited highest expression in *C. flexuosus*, followed by *C. winterianus* (80%) with negligible expression in *C. martinii*, which corroborated with the aldehyde (citral and CAL) content in respective species (**Figures 4B** and **5**). Similar to *CfADH1, CfADH2a* and *CfAKR2b*, expression of *CfCCD1* was consistent with the aldehyde content in leaf of *C. flexuosus* (**Figure 6**). As suggested for aldehyde formation in *O. basilicum* (Iijima et al., 2006), multiple enzymes (ADH1, ADH2a, AKR2b, and CCD1) could be responsible for differential accumulation of different aldehydes in *Cymbopogon* species.

Esterification of alcohol to corresponding acetates by AATs is an important conjugative step in essential oil biosynthetic pathway. These acetates form one of the most predominant volatile esters in essential oils of different aromatic grasses. The essential oil profile of *C. martinii* revealed ∼21% of GA in inflorescence (Supplementary Figure S7B). Among the three *AATs* analyzed, only *AAT3* exhibited significant expression in inflorescence and was high in *C. martinii* followed by *C. flexuosus* (∼48%) with negligible expression in *C. winterianus* (**Figure 5**). Also, the tissue specific expression (which accumulates high GA) indicated proportional expression with GA content (Supplementary Figures S7B,C). These results are consistent with previous reports of correlation of expression of acetyl-CoA:benzylalcohol acetyltransferase in *C. breweri* and *GAAT* in *R. hybrida* with floral scent acetates (Dudareva et al., 1998; Shalit et al., 2003). Hence, *AAT3* may be the possible candidate involved in GA formation in *Cymbopogon* sp. Among the 3 ALDH candidates, while *ALDH3* showed higher expression in all three species, ALDH1 and ALDH2 expression was negligible, implicating the possible role of ALDH3 in geranic/citronellic acid formation in *Cymbopogon* (Supplementary Figure S8B).

### Molecular Modeling and Docking

**FIGURE 7:**
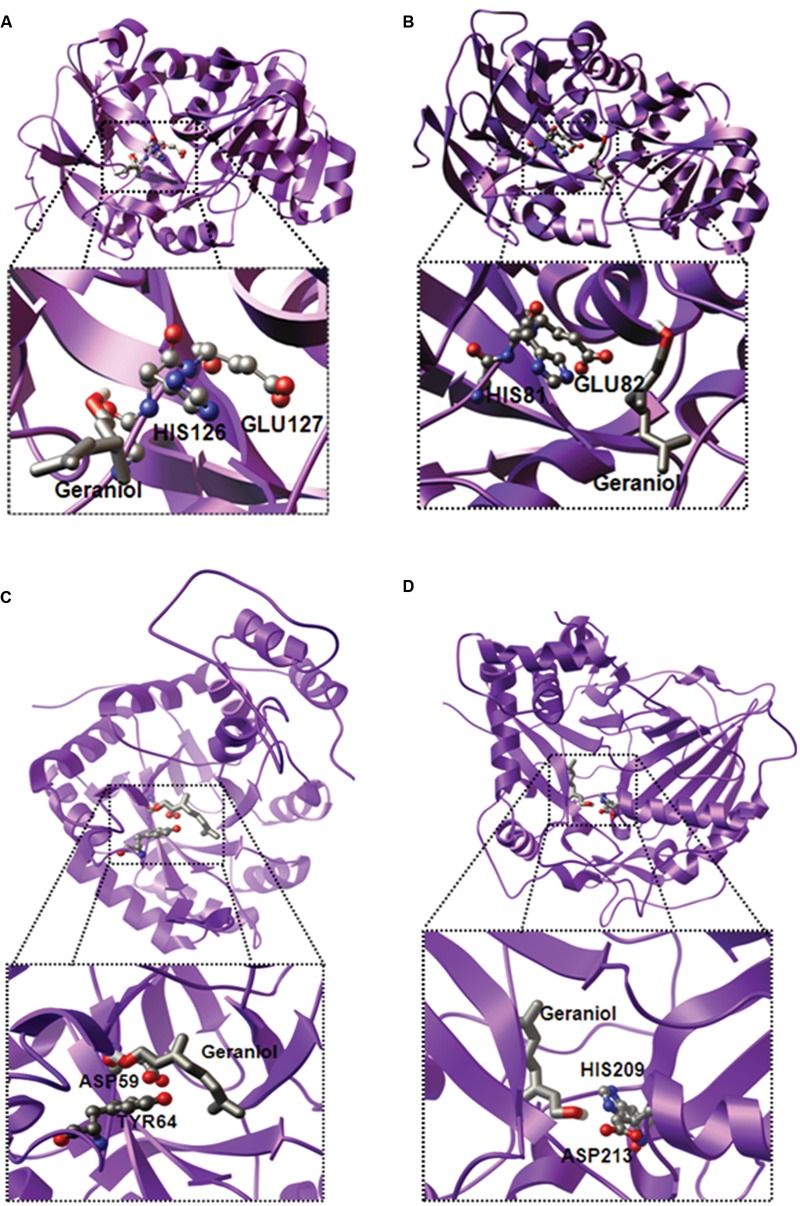
**Three-dimensional ribbon model of the complete structure of CfADH1 **(A)** CfADH2a **(B)**, CfAKR2b **(C)**, and CfAAT3 **(D)**.** The homology models of CfADH1 and 2a, CfAKR2b, CfAAT3, and CfALDH3 were built using the X-ray structures of *Populus tremuloides* synapyl alcohol dehydrogenase (PDB ID: 1YQD), *Rauvolfia serpentina* perakine reductase (PDB ID: 3V0T), and *Coffea canephora* hydroxycinnamoyl transferase (PDB ID: 4G0B), respectively. The substrate bound complexes were visualized by PMV software (http://mgltools.scripps.edu). The active site residues are represented as ball and stick, and substrates are shown as stick (colored by atom type).

Analysis of gene expression and essential oil profiling indicated the involvement of CfADH1, CfADH2a, CfAKR2b, CfAAT3, and CfALDH3 in *Cymbopogon* aroma biosynthesis. To further support the role of these candidates, *in silico* studies were performed to know the possible 3D structure and substrate interactions. The conversion of hydroxyl group to aldehyde requires a co-enzyme NAD^+^ and co-factor zinc (Zn^++^), and conserved catalytic motifs. While conserved glycine rich “GXGXXG” and catalytic “GHEXXGXXXXXGV” motifs are reported for ADH activity (Lüddeke et al., 2012; Iijima et al., 2014), AKR requires conserved “DXXXXY” motif having catalytic aspartic acid (D) and tyrosine (Y) for its enzyme function (Kavanagh et al., 2008). Sequence analysis revealed the presence of these conserved motifs in CfADH1, CfADH2a, and CfAKR2b (Supplementary Figures S10A,B and S11), suggesting their NAD-dependent dehydrogenase and aldo-keto reductase activity, respectively. Indeed, molecular docking data clearly demonstrated that the GOL binds very proximal to the conserved catalytic motifs of CfADH1, CfADH2a, and CfAKR2b proteins (**Figures 7A–C**; Supplementary Figure S10A–C and S11; Supplementary Table S6). Among the tested aliphatic and aromatic alcohols, GOL exhibited the lowest ΔG energy for all three proteins (Supplementary Table S6). Further, the docking revealed that the active site topology of CfADH1 and CfADH2b contains glutamic acid (E) and histidine (H) residues, where GOL binds and gets converted to GAL in presence of NAD^+^ (**Figures 7A,B**). The conserved “E” plays a vital role in ADH function, as it facilitates substrate binding and coordination of Zn^++^ ion during catalysis (Ryde, 1995). The GOL bound complex of CfAKR2b exhibited the ΔG energy of -6.96 kcal/mol (**Figure 7C**; Supplementary Table S6). It is possible that the co-factor NAD^+^ binds to the conserved sites in ADH and AKR where the reduction of NAD^+^ occurs by accepting hydrogen from GOL (Strommer, 2011). In the case of CfAAT3 also, docking studies demonstrated higher affinity of CfAAT3 for GOL (ΔG energy of -5.67 kcal/mol) along with farnesol (ΔG energy of -5.97 kcal/mol) among the tested aliphatic and aromatic alcohols (**Figure 7D** and Supplementary Table S6). The GOL-bound CfAAT3 complex revealed that the conserved histidine (H) and aspartic acid (D) of catalytic HXXXD motif are closely located in the active site (**Figure 7D** and Supplementary Figure S13), where GOL is converted to GA by acetyltransferase activity (Aharoni et al., 2000; D’Auria, 2006). Similarly for CfALDH3, the molecular modeling and docking studies revealed greater affinity (ΔG energy of -6.16 kcal/mol) for GAL-bound CfALDH3 complex (Supplementary Figures S9 and S14; Supplementary Table S6). Overall, the results from molecular modeling supported the gene expression data, further indicating the involvement of these candidates in essential oil formation. Nevertheless, further experimental evidences (i.e., site directed mutagenesis and *in vitro* assays) are needed to confirm their precise role in *Cymbopogon* aroma formation.

### SSR Mining and Distribution Analysis

**FIGURE 8:**
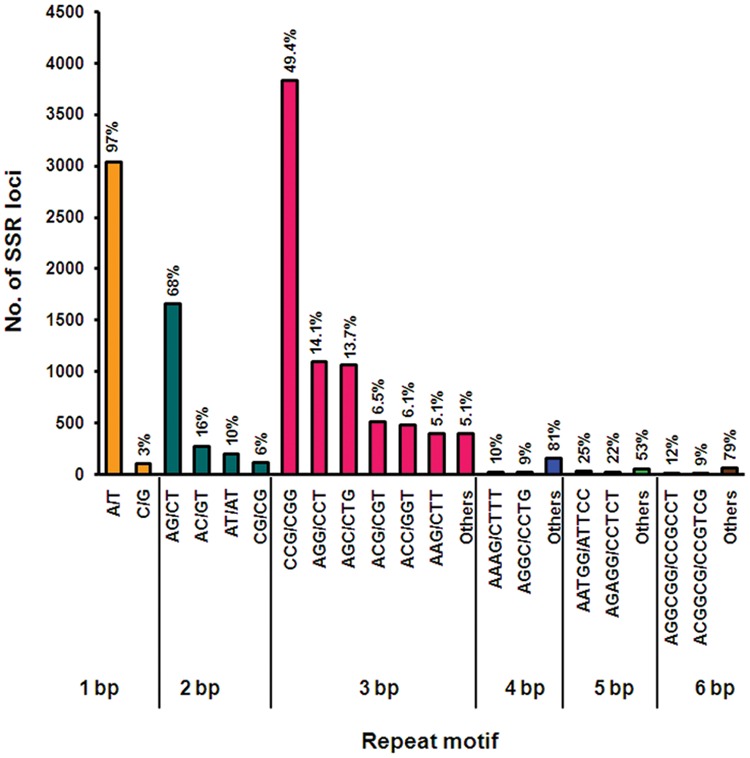
**Summary of SSR types identified in *C. flexuosus* transcriptome.** Total number of SSR loci in each repeat motif is represented in *Y*-axis. Percentage value in the graph indicates the share of individual repeat motif for each repeat type considering the total number in each repeat type as 100%.

Simple sequence repeats (SSRs) are tandem repeats of DNA sequences present in abundance and are distributed throughout the genome (Liu et al., 2013). They are highly versatile PCR-based markers, which are successfully used in marker-assisted selection (MAS), comparative genomics, genetic diversity, and evolutionary studies (Liu et al., 2013). Transcriptome SSR markers, also called as genic-SSRs or EST-SSRs, exhibit high inter-specific transferability as they are located in the coding region of the gene in contrast to genomic SSRs (Liu et al., 2013). Although several molecular markers consisting of RAPD, ISSR, and genomic SSRs have been developed (Khanuja et al., 2005; Kumar et al., 2007; Adhikari et al., 2015; Bishoyi et al., 2016), so far no genic-SSRs are available for *Cymbopogon*. In recent years, NGS technologies has massively increased the number of SSR markers discovered for both major crops and non-model plant species including *Lilium* (Shahin et al., 2012), *O. sativa* (Miah et al., 2013), and *Setaria viridis* (Xu et al., 2013). Here, for the first time we have mined genic/EST SSRs from lemongrass transcriptome that can be utilized for further crop improvement. Mining of assembled transcripts from *C. flexuosus* leaf transcriptome resulted in 10,715 (11.5%) transcripts containing 12,968 promising SSRs, of which 1,805 (16.8%) sequences contained >1 SSRs (Supplementary Table S7). A total of 966 SSRs were found to be present in compound formation (Supplementary Table S7). For the motif type, tri-nucleotide (59.8%) repeats were most abundant, followed by mono- (24.2%) and di- nucleotide repeats (13.1%) (**Figure 8** and Supplementary Table S7), which was consistent with earlier reports on other monocot species (Davey et al., 2013; Miah et al., 2013). The most common tri-nucleotide repeats were CCG/CGG in lemongrass (**Figure 8** and Supplementary Table S8), which was also previously reported for *Cymbopogon jwarancusa* using genomic library (Kumar et al., 2007). The abundance of CCG repeats has been observed as a special feature of monocot genomes attributed to their higher GC content (Morgante et al., 2002). The most common mono- and di-nucleotide repeats were A/T and AG/CT, respectively (**Figure 8** and Supplementary Table S8). Several SSR motifs were linked to transcripts of several secondary metabolic pathways including terpenoid pathways with 6 and 9 SSR motifs linked to MEP pathway and MVA pathway, respectively (Supplementary Tables S3 and S4). In addition, SSR motifs linked to genes involved in other pathways such as steroid, brassinosteroid, alkaloid and phenylpropanoid were also found (Supplementary Table S4). Among the short listed candidates possibly involved in essential oil biosynthesis, transcripts encoding CfPPase1, CfADH1, and CfADH2a contained (AG)6 and (GCC)5ggccgatccgccgcccggcgatgatg(CGT)5 at 5′ UTR, (A)19 at 5′ UTR, and (GGC)5 within the ORF, respectively. Similar to our observation, it has been previously reported that dinucleotide SSR motifs are located in the 5′ UTRs or in the introns of the genes, which are known to regulate promoter activity (Morgante and Olivieri, 1993; Debrauwere et al., 1997). The presence of trinucleotide motifs in the ORF of the genes can be attributed to the tolerance for frame shift mutations in coding regions (Richard and Dujon, 1997; Varshney et al., 2005). The genic/EST-SSRs generated in this study hold great potential for identifying functional markers and will aid in genetic and genomic studies in *Cymbopogon* as they are better tools than other markers because of their co-dominant inheritance, multi-allelic nature, and high reproducibility (Zhao et al., 2012). The role of these motifs in pathway genes containing SSRs needs to be further investigated in *Cymbopogon*.

## Conclusion

*Cymbopogon* is the most important essential oil producing aromatic grass of Poaceae family. The transcriptome resource for this economically important genus remains unexplored to date. Here, we have generated a gene catalog for lemongrass (*C. flexuosus*) using *de novo* transcriptome assembly, which led to the discovery of potential genes (*PPase, ADH, AKR, CCD, AAT*, and *ALDH*) involved in essential oil biosynthesis. Notably, comparative and tissue specific expression of identified genes correlated with the essential oil profiles of different *Cymbopogon* species, supporting their possible involvement in essential oil biosynthesis. In addition, molecular modeling of identified proteins supported the gene expression, thereby validating their role in essential oil biosynthesis. Biochemical and *in planta* functional characterization of identified candidates could unravel different steps of essential oil biosynthesis and regulation in *Cymbopogon*, which would be the subject of our future investigations. The putative SSR markers generated in this study could be used for association mapping and molecular breeding programs to modulate/improve essential oil profile/yield in aromatic grasses. This report, for the first time, provides transcriptomic insights into the essential oil biosynthesis of aromatic grass species. We anticipate that this work will take the research on *Cymbopogon* to the next level, facilitating characterization of genes, regulators and functional markers, and also engineering of essential oil biosynthetic pathway in aromatic grasses or through synthetic biology approaches.

## Author Contributions

Conceived and designed the experiment: DN and DR. Performed the experiments: SM, SK, DR, VD, HS, and SR. Contributed reagents/materials/analysis tools: AS. Wrote the paper: SM, SK, DR, and DN.

## Conflict of Interest Statement

The authors declare that the research was conducted in the absence of any commercial or financial relationships that could be construed as a potential conflict of interest.

## Footnotes

1 https://blast.ncbi.nlm.nih.gov/Blast.cgi

2 UniProtKB, http://www.uniprot.org/blast/

3 http://www.genome.jp/kaas-bin/kaas_main?mode=partial

4 http://pgrc.ipk-gatersleben.de/misa/

5 http://web.expasy.org/translate

6 http://mafft.cbrc.jp/alignment/software/

7 http://www.ch.embnet.org/software/BOX_form.html

8 http://www.megasoftware.net

9 www.rcsb.org">http://www.rcsb.org">www.rcsb.org

10 http://www.shigen.nig.ac.jp/rice/oryzabase

11 http://rice.plantbiology.msu.edu/

12 http://phytozome.jgi.doe.gov

13 http://www.plantgdb.org/
