# Supplementary material for: De Novo Sequencing and Analysis of Lemongrass Transcriptome Provide First Insights into the Essential Oil Biosynthesis of Aromatic Grasses
Source: Front Plant Sci. 2016 Jul 28;7:1129. doi: 10.3389/fpls.2016.01129 (PMC4963619; doi:10.3389/fpls.2016.01129)
Supplement: Supplementary file 1 [file Presentation_1.PDF]

Supplementary material

De novo Sequencing and Analysis of Lemongrass Transcriptome Provide First Insights into the Essential Oil Biosynthesis of Aromatic Grasses

Seema Meena[1,#], Sarma Rajeev Kumar[1,#], D.K. Venkata Rao[1], Varun Dwivedi[1], H.B. Shilpashree[1], Shubhra Rastogi[2], Ajit Kumar Shasany[2], and Dinesh A. Nagegowda[1*]

[*]Correspondence: Dr. Dinesh A. Nagegowda, da.nagegowda@cimap.res.in

Table S1 List of qRT-PCR primers used in the study

CfTPS1 RT F	GATTCCATTCGCCCTTTGTC
CfTPS1 RT R	AGGCCCTCCTGGAAATCAA
CfPP1 RT F	CATCAAGCAGGACACCAAGAAG
CfPP1 RT R	CCATGTTTGAGGAAGTAACCCATA
Cf PP2 RT F	ATGATTGACCAGGGTGAAAAGG
Cf PP2 RT R	ATGTCGTTGTAGTGACGGTATTCAG
CfADH1 RT F	GAGTTGGTTCTTAATTTGTCATGGTTT
CfADH1 RT R	CGGCCCTGTCTCCCTCTTAT
CfADH2a RT F	CACGGCCTCTGCAAGCA
CfADH2a RT R	GCAGGCCAACCAAGATCATC
CfADH3b RT F	GGACACCTCTCCCCCTACAC
CfADH3b RT R	CAGATCCCACAGTAGAGC
CfADH4 RT F	GCTCGGAAAACAAAAAGGATGA
CfADH4 RT R	TCTTGGTCGTCGCTGTTAACA
CfAKR1 RT F	GCATCGACCTCTACTACCAGCAT
CfAKR1 RT R	GCTCACCCATCGTGATCTCA
CfAKR2a RT F	CGACCGTTATGCTGGAATTA
CfAKR2a RT R	TGGCTACTCATATTTCCAAGAAGA
CfAKR2b RT F	CAGATGAGATGGCCGAACTTG
CfAKR2b RT R	GGTGTTGGCCATTTGAGGAT
CfCCD1 RT F	AAACACTCTTTCACTGCTCATCCA
CfCCD1 RT R	GGAGGTTCATGCGAATATCCA
CfAAT1 RT F	CTATGAAGGGCTGGCGTTTG
CfAAT1 RT R	GCTCAGCCTGCAATGAGATG
CfAAT2 RT F	CGATGCAGCAGCGTCAGT
CfAAT2 RT R	GCACGAACCAGACAAGAAAGTG
CfAAT3 RT F	CAACCGCTCGAAACCAACA
CfAAT3 RT R	CACGCCCATGAAGTTTGGA
CfALDH1 RT F	TTGCTGTGCTGGGTCTCGTA
CfALDH1 RT R	GGCCTTCTCCACGAACTCATC
CfALDH2 RT F	ATCCCCATCGCCATTGCT
CfALDH2 RT R	AGACATCACGCTGACAAGTGACA
CfALDH3 RT F	TGGGCCTGTTCAGTCAATCC
CfALDH3 RT R	CCAATCCATACTGGCTTGCA
EF1a RT F	TCTCGGAGCTGCTCACCAA
EF1a RT R	GTCGCCATTCTTGAGGAACTTG
Cf Actin RT F	GACTACGACCAGGAGATGGAGACT
Cf Actin RT R	ATGACCTGTCCATCAGGAAGCT
Cf GAPDH RT F	CCCGACGAGCCCATCAT
Cf GAPDH RT R	CTTTTGGTCGAGCACCTTGAC

Table S2 Annotation summary of *C. flexuosus transcripts*

Database	Total no. of annotated transcripts	Percentage
NCBI	76293	82.80%
Uniprot	69984	75.95%
KEGG	24147	26.21%
AGRIS	5867	6.37%
**Total**	**92139**	**100.00%**

Table S3 Aroma biosynthetic pathway genes identified in *C. flexuosus* transcriptome and SSRs associated with them.

Gene	No. of trans-cripts	Avg. FPKM	No. of transcrip ts (SSR-linked)	No. of SSRs	SSR motifs
**MEP pathway genes**					
1-Deoxy-D-xylulose 5-phosphate synthase (DXS)	21	4.29	3	2	(TG)7 and (CTC)5
1-Deoxy-d-xylulose 5-phosphate reducto isomerase (DXR)	4	2.85	-	-	-
2-C-methyl-D-erythritol 4-phosphate cytidylyl transferase (MCT)	3	3.76	-	-	-
4-Diphosphocytidyl-2-C-methyl-D-erythritol kinase (CMK)	5	15.88	-	-	-
2-C-methyl-D-erythritol 2,4-cyclodiphosphate synthase (ispF)	1	20.42	-	-	-
4-Hydroxy-3-methylbut-2-enyl diphosphate synthase (HDS)	11	10.47	-	-	-
4-hydroxy-3-methylbut-2-enyl diphosphate reductase (HDR)	11	22.70	7	1	(CAC)6
**MVA pathway genes**					
Acetoacetyl-CoA thiolase/acetyl-CoA C-acetyltransferase (AACT)	16	8.22	1	1	(CCG)6
3-Hydroxy-3-methylglutaryl coenzyme A synthase (HMGS)	21	3.56	1	1	(CGG)8
3-Hydroxy-3-methylglutaryl-coenzyme A reductase (HMGR)	25	7.88	7	5	(A)10, (T)12, (AGC)5, (CGC)5 and (TCC)5
Mevalonate kinase (MVK)	3	2.73	-	-	-
5-Phosphomevalonate kinase (PMK)	8	5.17	-	-	-
Mevalonate diphosphate decarboxylase (MVD)	4	3.55	-	-	-
**Downstream terpene pathway genes**					
Isopentenyl-diphosphate isomerase (IDI)	9	17.73	4	2	(C)23 and (GCA)5
Geranyl diphosphate synthase (GDS)	8	2.96	-	-	-

Farnesyl diphosphate synthase (FPPS)	9	3.65	2	2	(TC)6 and (CGC)6
Geranylgeranyl diphosphate synthase (GGDS)	5	6.98	4	3	(AT)6, (CGG)5 and (CAC)6
Geranylgeranyl diphosphate synthase small subunit (GGDS.SSU)	4	2.64	-	-	-
**Aroma biosynthetic genes identified in *C. flexuosus***					
Terpene synthase (TPS)	16	3.087	1	1	(T)11
Pyrophosphatase (PPase)	19	78.96	3	7	(G)26, (AG)6, (CCG)5 and (GCC)5ggccgatccgccgcccggc gatgatg(CGT)5
Nudix hydrolase (NUDX)	26	6.06	4	5	(GA)7, (GCG)5, (CGG)7, (CGC)7 and (CGAT)5
Alcohol dehydrogenases (ADH)	92	22.72	21	8	(A)16/19/35, (T)10, (TC)9, (GGC)5/8, (GCC)5, (CGC)6 and (GGA)5gaagagggaggagccgaa ggctggggcggaggcgggggcaggg gagga(GGC)8/9
Aldo-Keto Reductases (AKR)	38	7.70	5	3	(A)11, (GGC)5 and (CGG)7
Carotenoid Cleavage Dioxygenases (CCD)	11	17.62	-	-	-
Alcohol acyltransferases (AAT)	35	6.76	8	3	(T)10/13, (GTC)6 and (GGCA)5
Aldehyde dehydrogenases (ALDH)	88	16.67	6	4	(TCA)5, (GCC)5, (CAG)5 and (CCTCA)5

Table S4 Other important secondary metabolic pathway genes identified in *C. flexuosus* transcriptome and SSRs putatively associated with them.

Gene	No. of transcripts	Avg. FPKM	No. of transcripts(SSR-linked	No. of SSRs	SSR motifs
**Phenylpropanoid, flavanoid and anthocyanin biosynthetic pathway genes**					
Phenylalanine ammonia-lyase (PAL)	59	33.55	2	2	(A)10 and (CTC)6
Cinnamate 4-hydroxylase (C4H)	10	8.19	-	-	-
beta-glucosidase (bgl)	37	11.59	5	4	(A)11/12, (GCG)5 and (GCG)5agcagaacaacgcagcagatgaacaccaccctcgccggcgtcatgac(CG)
Catalase-peroxidase (KatG)	3	2.85	-	-	-
4-Coumarate:coenzyme A ligase (4CL)	9	5.03	2	1	(CGA)5
Cinnamoyl-CoA reductase (CCR)	16	6.63	1	1	(CAA)5
Cinnamyl alcohol dehydrogenase (CAD)	24	9.11	13	6	(A)19, (GGC/GCC)5/8, (CGC)6 and (GGA)5gaagagggaggagccgaaggctggggcggaggcgggggcaggggagga(GGC)8
shikimate O-hydroxycinnamoyltransferase (HCT)	4	9.21	-	-	-
coumaroylquinate(coumaroylshikimate) 3'-monooxygenase (C3'H)	1	5.84	-	-	-
caffeoylshikimate esterase (CSE))	1	1.85	-	-	-
Caffeic acid 3-O-methyltransferase (COMT)	6	41.95	2	2	(GA)7 and (TGC)5
Caffeoyl CoA O-methyltransferase (CCOMT)	8	19.57	-	-	-
Chalcone synthase (CHS)	2	3.19	-	-	-
Chalconeisomerase (CHI)	14	20.33	2	1	(GGC)5
Flavanol synthase(FLS)	3	4.01	-	-	-
Flavonoid 3'-hydroxylase(F3'H)	6	9.63	4	5	(A)11 and (ACG)5

Dihydroflavonol 4-reductase (DFR)	3	3.53	-	-	-
Flavonoid 3',5'-hydroxylase (CYP75A)	5	12.52	4	1	(T)10
Leucoanthocyanidindioxygenase (ANS)	2	2.25	-	-	-
Leucoanthocyanidinreductase (LAR)	1	2.84	-	-	-
Anthocyanidinreductase (ANR)	8	20.14	-	-	-
Isoflavone 7-O-glucoside-6"-O-malonyltransferase (IF7MAT)	1	5.33	-	-	-
Flavonol-3-O-beta-D-glucoside 6"-O-malonyltransferase	5	2.48	3	2	(GCC)5 and (GGCA)6
beta-glucuronidase (GUSB)	6	5.14	3	1	(TAA)5
UDP-glucose:flavonol 3-O-glucosyltransferase	2	2.24	-	-	-
UDP-glucose:flavonoid 7-O-glucosyltransferase	1	1.81	-	-	-
Querceitin/flavonoid/flavonol 3-O-methyltransferase	2	17.38	2	3	(GA)7 and (TA)7
Flavonol 3-sulfotransferase	1	1.49	-	-	-
trans-resveratrol di-O-methyltransferase (ROMT)	2	4.70	-	-	-
Quinate O-Hydroxycinnamoyltransferase	3	2.91	-	-	-
Anthocyanidin 5,3-O-glucosyltransferase (GT1)	8	3.64	-	-	-
Aanthocyanin 5-aromatic acyltransferase (5AT)	1	2.38	-	-	-
**Alkaloid biosynthetic pathway genes**					
Strictosidine synthase	15	10.00	3	1	(CGC)5
Aspartate aminotransferase (GOT/ASP)	19	16.26	5	4	(T)10/15, (A)18 and (CTC)6
Tyrosine aminotransferase (TAT)	2	5.05	-	-	
**Taxol biosynthetic pathway genes**					
5-alpha-taxadienol-10-beta-hydroxylase	5	3.00	-	-	-
Taxadien-5-alpha-ol O-acetyltransferase	5	6.00	-	-	-

10-deacetylbaccatin III-10-O-acetyl transferase-like	1	2.90	-	-	-
3-N-debenzoyl-2-deoxytaxol N-benzoyltransferase	7	6.90	-	-	-
**Steroid biosynthetic pathway genes**					
Squalene synthase (SQS)	8	3.32	-	-	-
Squalenemonooxygenase (SQE)	4	2.57	-	-	-
Cycloartenol synthase	2	6.55	-	-	-
Delta24-sterol reductase (DHCR24)	10	17.93	1	1	(T)12
Sterol 14-demethylase (CYP51)	2	5.21	-	-	-
Sterol 24-C-methyltransferase (SMT1)	6	12.60	2	2	(AC)6 and (CCG)5
Delta14-sterol reductase (TM7SF2)	2	4.88	1	1	(A)10
Methylsterolmonooxygenase (SMO)	3	6.78	-	-	-
Cycloeucalenolcycloisomerase (CPI1)	6	5.52	-	-	-
Sterol-4alpha-carboxylate 3-dehydrogenase (NSDHL)	3	2.97	-	-	-
Cholestenol delta-isomerase (EBP)	6	5.64	3	2	(A)10 and (TC)7
24-methylenesterol C-methyltransferase (SMT2)	7	25.58	7	2	(TGG)5 and (GAG)5/6/7
Lathosterol oxidase (SC5DL)	1	7.39	-	-	-
Delta7-sterol reductase (DHCR7)	8	6.83	1	1	(A)12
CYP710A	1	2.09	-	-	-
Sterol O-acyltransferase (SOAT)	8	3.51	1	1	(CGC)5cggtgacgctcccgctccgcagcagcagcaggagcagaggcatcagg(AGC)5
3-beta-hydroxy-delta5-steroid dehydrogenase (3β-HSD)	14	2.75	-	-	-
**Brassinosteroid biosynthetic pathway genes**					
Steroid 22-alpha-hydroxylase (CYP90B1/DWF4)	4	2.66	-	-	-
CYP90A1 (CPD)	2	9.74	-	-	-
Steroid 5-alpha-reductase (DET2)	2	1.28	-	-	-
Brassinosteroid-6-oxidase 1/2 (CYP85A1/A2/;BR6OX1/2)	4	8.82	4	5	(GAA)5, (GGA)6 and (TCT)5
CYP734A4	3	2.38	-	-	-

Table S5 Identity and similarity matrix of the identified *C. flexuosus* candidates with other characterized proteins

***C. flexuosus* candidates**	**Homology with the characterized Genes**								
**Terpene synthase**	**VvLIS/NES (AEY82696)**	**VvGES (ADR74218)**							
**CfTPS1**	I=42%, S=62%, QC=92%	I=36%, S=57%, QC=86%							
**Alcohol dehydrogenase**	**AaCAD1 (ACB54931)**	**CdGeDH (WP_043683915)**	**ClGeDH (B2NI93)**	**ObCAD1 (Q2KNL5)**	**ObGeDH (Q2KNL6)**	**PcGeDH (AFY63473)**	**PfGeDH (AFY63472)**	**PsGeDH (AFY63474)**	**ZoGeDH1 (BAR42579)**
**CfADH1**	I=51%, S=68%, QC=82%	I=29%, S=42%, QC=75%	I=26%, S=43%, QC=91%	I=51%, S=69%, QC=83%	I=56%, S=72%, QC=83%	I=57%, S=74%, QC=83%	I=57%, S=74%, QC=83%	I=57%, S=74%, QC=83%	I=59%, S=75%, QC=87%
**CfADH2a**	I=45%, S=63%, QC=95%	I=28%, S=42%, QC=86%	I=23%, S=38%, QC=88%	I=46%, S=65%, QC=93%	I=53%, S=72%, QC=96%	I=54%, S=71%, QC=96%	I=54%, S=71%, QC=96%	I=54%, S=71%, QC=96%	I=65%, S=77%, QC=96%
**CfADH2b**	I=47%, S=65%, QC=93%	I=29%, S=42%, QC=85%	I=24%, S=41%, QC=73%	I=48%, S=67%, QC=92%	I=53%, S=71%, QC=94%	I=56%, S=73%, QC=94%	I=56%, S=73%, QC=94%	I=56%, S=73%, QC=94%	I=62%, S=76%, QC=94%
**CfADH3a**	I=75%, S=84%, QC=98%	I=35%, S=82%, QC=4%	I=25%, S=38%, QC=90%	I=78%, S=86%, QC=97%	I=48%, S=67%, QC=95%	I=50%, S=68%, QC=95%	I=50%, S=68%, QC=95%	I=50%, S=68%, QC=95%	I=49%, S=67%, QC=95%
**CfADH3b**	I=75%, S=84%, QC=98%	I=35%, S=82%, QC=4%	I=25%, S=38%, QC=90%	I=78%, S=85%, QC=97%	I=49%, S=67%, QC=94%	I=50%, S=68%, QC=95%	I=50%, S=68%, QC=95%	I=50%, S=68%, QC=95%	I=49%, S=66%, QC=95%
**CfADH3c**	I=74%, S=83%, QC=98%	I=29%, S=54%, QC=8%	I=25%, S=38%, QC=91%	I=78%, S=85%, QC=97%	I=50%, S=67%, QC=94%	I=51%, S=69%, QC=94%	I=51%, S=69%, QC=94%	I=51%, S=69%, QC=94%	I=49%, S=66%, QC=94%
**CfADH3d**	I=73%, S=84%, QC=98%	I=29%, S=54%, QC=8%	I=25%, S=38%, QC=91%	I=77%, S=85%, QC=97%	I=50%, S=68%, QC=94%	I=51%, S=69%, QC=94%	I=51%, S=69%, QC=94%	I=51%, S=69%, QC=94%	I=49%, S=67%, QC=94%

**CfADH3e**	I=74%, S=83%, QC=98%	I=29%, S=54%, QC=8%	I=25%, S=38%, QC=91%	I=77%, S=85%, QC=97%	I=50%, S=67%, QC=93%	I=51%, S=69%, QC=93%	I=51%, S=69%, QC=93%	I=51%, S=69%, QC=93%	I=49%, S=66%, QC=94%
**CfADH4**	I=23%, S=37%, QC=87%	I=31%, S=47%, QC=95%	I=35%, S=52%, QC=97%	I=23%, S=38%, QC=92%	I=25%, S=40%, QC=91%	I=25%, S=39%, QC=88%	I=25%, S=39%, QC=88%	I=25%, S=39%, QC=88%	I=24%, S=43%, QC=81%
**Aldo-keto reductase**	**BdAKR (XP_003575318)**	**ObAKR (XP_006652179)**	**PcAKR (AFV99149)**	**PfAKR (AFV99148)**	**PsAKR (AFV99150)**				
**CfAKR1**	I=76%, S=86%, QC=97%	I=80%, S=88%, QC=96%	I=62%, S=75%, QC=98%	I=61%, S=74%, QC=98%	I=61%, S=74%, QC=98%				
**CfAKR2a**	I=82%, S=90%, QC=99%	I=87%, S=93%, QC=100%	I=68%, S=80%, QC=99%	I=67%, S=80%, QC=99%	I=67%, S=80%, QC=99%				
**CfAKR2b**	I=82%, S=90%, QC=99%	I=89%, S=95%, QC=100%	I=68%, S=81%, QC=99%	I=68%, S=81%, QC=99%	I=68%, S=80%, QC=99%				
**Carotenoid cleavage dioxygenase**	**OsCCD1 (AK066766)**	**SlCCD1A (AAT68187)**	**SlCCD1B (AAT68188)**						
CfCCD1	I=99%, S=99%, QC=100%	I=78%, S=86%, QC=98%	I=74%, S=86%, QC=98%						
**Alcohol acyl transferase**	**CbBEBT (AAN09796)**	**FaAAT2 (AEM43830)**	**FaSAAT (AAG13130)**	**FcAAT1 (ACT82247)**	**FvAAT (AAN07090)**	**MsAAT1 (CAC09063)**	**RhAAT (AAW31948)**		
CfAAT1	I=30%, S=47%, QC=97%	I=28%, S=49%, QC=85%	I=23%, S=41%, QC=91%	I=23%, S=40%, QC=90%	I=24%, S=42%, QC=91%	I=28%, S=45%, QC=97%	I=23%, S=40%, QC=85%		
CfAAT2	I=33%, S=48%, QC=71%	I=31%, S=45%, QC=72%	I=26%, S=46%, QC=40%	I=28%, S=45%, QC=39%	I=26%, S=44%, QC=33%	I=40%, S=57%, QC=98%	I=23%, S=38%, QC=73%		
CfAAT3	I=26%, S=35%, QC=75%	I=24%, S=38%, QC=59%	I=22%, S=36%, QC=90%	I=22%, S=38%, QC=97%	I=23%, S=38%, QC=89%	I=28%, S=38%, QC=60%	I=22%, S=37%, QC=96%		

Aldehyde dehydrogena se	AmBALHD (ACM89738)	AtALDH2b (Q8S528.2)	CdGalDH (CCF55023)	NtALDH (CAA71003)	OsALDH2b (AAF73828)	ZmRF2A (AAC49371)	ZmRF2B (AAL99613)	
CfALDH1	I=77%, S=85%, QC=99%	I=81%, S=90%, QC=91%	I=37%, S=55%, QC=86%	I=79%, S=90%, QC=91%	I=99%, S=99%, QC=100%	I=90%, S=94%, QC=99%	I=76%, S=86%, QC=100%	
CfALDH2	I=72%, S=83%, QC=100%	I=76%, S=87%, QC=89%	I=37%, S=53%, QC=85%	I=76%, S=87%, QC=90%	I=80%, S=88%, QC=94%	I=78%, S=86%, QC=99%	I=84%, S=92%, QC=99%	
CfALDH3	I=80%, S=87%, QC=94%	I=81%, S=89%, QC=92%	I=38%, S=56%, QC=86%	I=81%, S=91%, QC=91%	I=92%, S=96%, QC=94%	I=94%, S=96%, QC=100%	I=77%, S=86%, QC=99%	

Table S6 Summary of molecular docking analyses of candidate proteins

Predicted polypeptides from *C. flexuosus*	Substrate	ΔG kcal/mol
CfADH1	**Geraniol**	**-6.17**
(Grid space, 0.619 ; grid	1-Octanol	-4.12
dimension (xyz, 57.579 124.	Farnesol	-5.60
528 35.04),	Cinnamyl alcohol	-3.22
	Sinnapyl alcohol	-3.25
CfADH2a	**Geraniol**	**-6.88**
(Grid space, 0.597; grid center	1-Octanol	-4.22
(xyz, 56.405,123.318, 29.928)	Farnesol	-4.99
	Cinnamyl alcohol	-3.22
	Sinnapyl alcohol	-2.99
CfAKR2b	**Geraniol**	**-6.96**
(Grid space, 0.35; grid center	1-Octanol	-3.99
(xyz,16.394 -1.929 19.407)	Farnesol	-4.33
	Cinnamyl alcohol	-5.12
	Sinnapyl alcohol	-4.32
CfAAT3	**Geraniol**	**-5.67**
Grid space, 0.525; grid center	1-Octanol	-3.12
(xyz, -42.258 -20.514 20.781)	Farnesol	-5.96
	Cinnamyl alcohol	-4.15
	Sinnapyl alcohol	-4.13
CfALDH3	**Geranial**	**-6.16**
(Grid space, 0.547 ; grid center	1-Octanal	-3.66
(xyz, 52.25 112.178 5.182)	Farnesal	-4.11
	Cinnamaldehyde	-2.66
	Sinapaldehyde	-2.16

Table S7 Summary of SSR analysis in *C. flexuosus* transcriptome

**SSR mining**	
Total number of sequences examined	92937
Total number of identified SSRs	12968
Number of SSR containing sequences	10715
Number of sequences containing more than 1 SSR	1805
Number of SSRs present in compound formation	966
**Distribution of SSRs in Different repeat types**	
Mono-nucleotide	3139
Di-nucleotide	1705
Tri-nucleotide	7755
Tetra-nucleotide	188
Penta-nucleotide	101
Hexa-nucleotide	80

Table S8 Summary of the number of classified repeat types considering sequence complementary in *C. flexuosus*

Repeat motif	No. of repeats							
	5	6	7	8	9	10	>10	Total
**Mono-nucleotide (3139, 24.2 %)**								
A/T	-	-	-	-	-	1314	1724	3038
C/G	-	-	-	-	-	30	71	101
**Di-nucleotide (1 705, 13.1 %)**								
AG/CT	-	305	210	138	116	81	305	1155
AC/GT	-	124	60	25	18	7	39	273
AT/AT	-	78	33	21	12	4	16	164
CG/CG	-	83	18	7	5	-	-	113
**Tri-nucleotide (7755, 59.8 %)**								
CCG/CGG	2226	987	364	174	64	16	1	3832
AGG/CCT	621	274	114	49	26	6	-	1090
AGC/CTG	581	295	110	55	14	2	4	1061
ACC/GGT	305	113	33	10	4	10	-	507
ACG/CGT	320	122	51	9	1	-	4	475
ACT/AGT	33	12	10	1	-	1	-	394
AGC/CTG	581	295	110	55	14	2	4	210
AGG/CCT	621	274	114	49	26	6	-	100
ATC/ATG	152	45	11	2	-	-	-	57
CCG/CGG	2226	987	364	174	64	16	1	29
**Tetra-nucleotide (188, 1.4 %)**								
AAAG/CTTT	11	1	5	2	-	-	-	19
AGGC/CCTG	14	3	-	-	-	-	-	17
AAAT/ATTT	5	8	-	-	-	-	-	13
ATCC/ATGG	10	-	3	-	-	-	-	13
ATCG/ATCG	13	-	-	-	-	-	-	13
AACC/GGTT	3	-	1	6	2	-	-	12
AATC/ATTG	12	-	-	-	-	-	-	12
AGCG/CGCT	11	-	-	-	-	-	-	11
AAGG/CCTT	5	3	-	-	-	-	-	8
ACTC/AGTG	7	-	-	-	-	-	-	7
AGAT/ATCT	7	-	-	-	-	-	-	7
AGCT/AGCT	7	-	-	-	-	-	-	7
ACAT/ATGT	5	-	-	-	-	-	-	5
ACGC/CGTG	2	3	-	-	-	-	-	5
AGCC/CTGG	5	-	-	-	-	-	-	5
ATGC/ATGC	5	-	-	-	-	-	-	5

AAAC/GTTT	4	-	-	-	-	-	-	4
AATT/AATT	3	1	-	-	-	-	-	4
ACAG/CTGT	3	1	-	-	-	-	-	4
AGGG/CCCT	4	-	-	-	-	-	-	4
AATG/ATTC	3	-	-	-	-	-	-	3
AACT/AGTT		-	2	-	-	-	-	2
ACCT/AGGT	1	-	1	-	-	-	-	2
CCGG/CCGG	2	-	-	-	-	-	-	2
AACG/CGTT	1	-	-	-	-	-	-	1
AAGC/CTTG	1	-	-	-	-	-	-	1
ACGG/CCGT	1	-	-	-	-	-	-	1
ACTG/AGTC	1	-	-	-	-	-	-	1
**Penta-nucleotide (101, 0.8 %)**								
AAAAC/GTTTT	1	-	-	1	-	-	-	25
AAAAG/CTTTT	1	1	-	-	-	-	-	22
AAATC/ATTTG	2	-	-	-	-	-	-	8
AACAC/GTGTT	7	-	-	-	-	-	-	7
AACGG/CCGTT	4	-	-	-	-	-	-	7
AACTG/AGTTC	-	1	-	-	-	-	-	4
AAGAG/CTCTT	5	3	-	-	-	-	-	4
AAGCT/AGCTT	3	-	-	-	-	-	-	4
AAGGG/CCCTT	3	4	-	-	-	-	-	3
AATCC/ATTGG	2	2	-	-	-	-	-	3
AATCG/ATTCG	1	-	-	-	-	-	-	2
AATGG/ATTCC	18	6	-	-	-	-	-	2
ACACG/CGTGT	1	-	-	-	-	-	-	2
ACAGT/ACTGT	1	-	-	-	-	-	-	1
ACATC/ATGTG	1	-	-	-	-	-	-	1
ACCTC/AGGTG	3	-	-	-	-	-	-	1
AGAGC/CTCTG	2	-	-	-	-	-	-	1
AGAGG/CCTCT	14	8	-	-	-	-	-	1
AGCAT/ATGCT	1	-	-	-	-	-	-	1
AGGGG/CCCCT	4	-	-	-	-	-	-	1
ATCCC/ATGGG	1	-	-	-	-	-	-	1
**Hexa-nucleotide (80, 0.6 %)**								
AAAAAC/GTTTTT	1	-	-	-	-	-	-	10
AAAAAT/ATTTTT	1	-	-	-	-	-	-	7
AAAACC/GGTTTT	1	-	-	-	-	-	-	6
AAAAGG/CCTTTT	3	-	-	-	-	-	-	5
AAATCC/ATTTGG	1	-	-	-	-	-	-	5
AACACC/GGTGTT	1	-	-	-	-	-	-	4
AACGCC/CGTTGG	2	-	-	-	-	-	-	4
AAGATG/ATCTTC	2	-	3	-	-	-	-	3

AAGGAG/CCTTCT	6	-	-	-	-	-	-	3
AATCCT/AGGATT	1	-	-	-	-	-	-	3
AATGAC/ATTGTC	1	2	-	-	-	-	-	2
ACAGCC/CTGTGG	-	1	-	-	-	-	-	2
ACATAT/ATATGT	3	1	-	-	-	-	-	2
ACCATC/ATGGTG	1	-	-	-	-	-	-	2
ACCGCC/CGGTGG	4	-	-	-	-	-	-	2
ACCTCC/AGGTGG	2	-	-	-	-	-	-	2
ACCTCG/AGGTCG	1	-	-	-	-	-	-	2
ACGAGG/CCTCGT	1	-	-	-	-	-	-	1
ACGATG/ATCGTC	-	1	-	-	-	-	-	1
ACGCCC/CGTGGG	1	-	-	-	-	-	-	1
ACGGCC/CCGTGG	2	-	-	-	-	-	-	1
ACGGCG/CCGTCG	7	-	-	-	-	-	-	1
AGAGGC/CCTCTG	2	-	-	-	-	-	-	1
AGAGGG/CCCTCT	1	-	-	-	-	-	-	1
AGATGG/ATCTCC	2	-	-	-	-	-	-	1
AGCATC/ATGCTG	1	2	-	-	-	-	-	1
AGCCGG/CCGGCT	5	-	-	-	-	-	-	1
AGCGGG/CCCGCT	1	-	-	-	-	-	-	1
AGGCCC/CCTGGG	2	-	-	-	-	-	-	1
AGGCGG/CCGCCT	10	-	-	-	-	-	-	1
AGGGCG/CCCTCG	1	-	-	-	-	-	-	1
ATATCC/ATATGG	1	-	-	-	-	-	-	1
ATCGGC/ATGCCG	2	-	-	-	-	-	-	1
**Total**	**4824**	**2607**	**1082**	**518**	**285**	**1481**	**2171**	**12968**
**%**	**37.2**	**20.1**	**8.3**	**4.0**	**2.2**	**11.4**	**16.7**	**100**

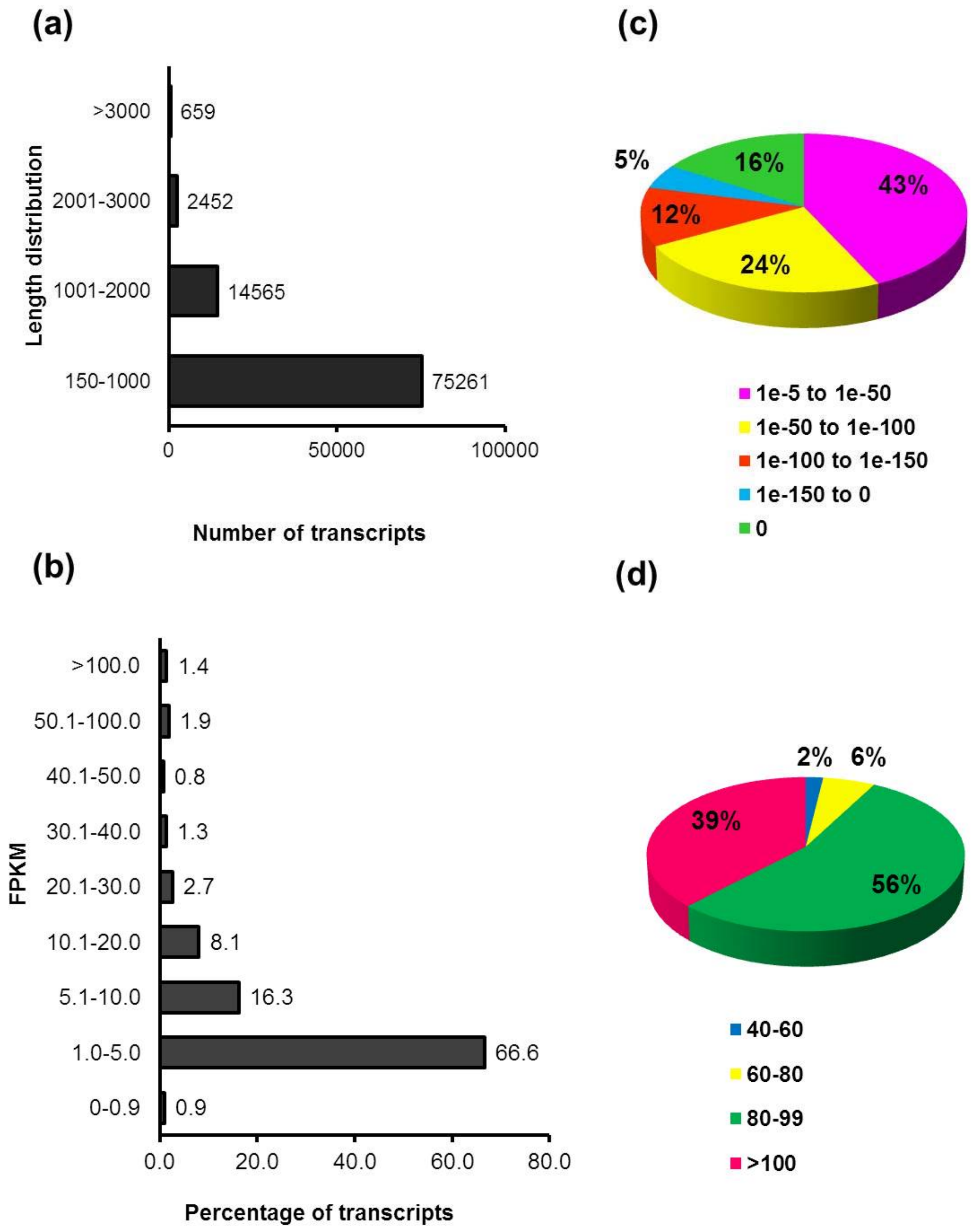

Figure S1 Length (a) and FPKM (b) distribution of assembled transcripts, and E-value (c) and similarity score (d) of the NCBI annotated transcripts.

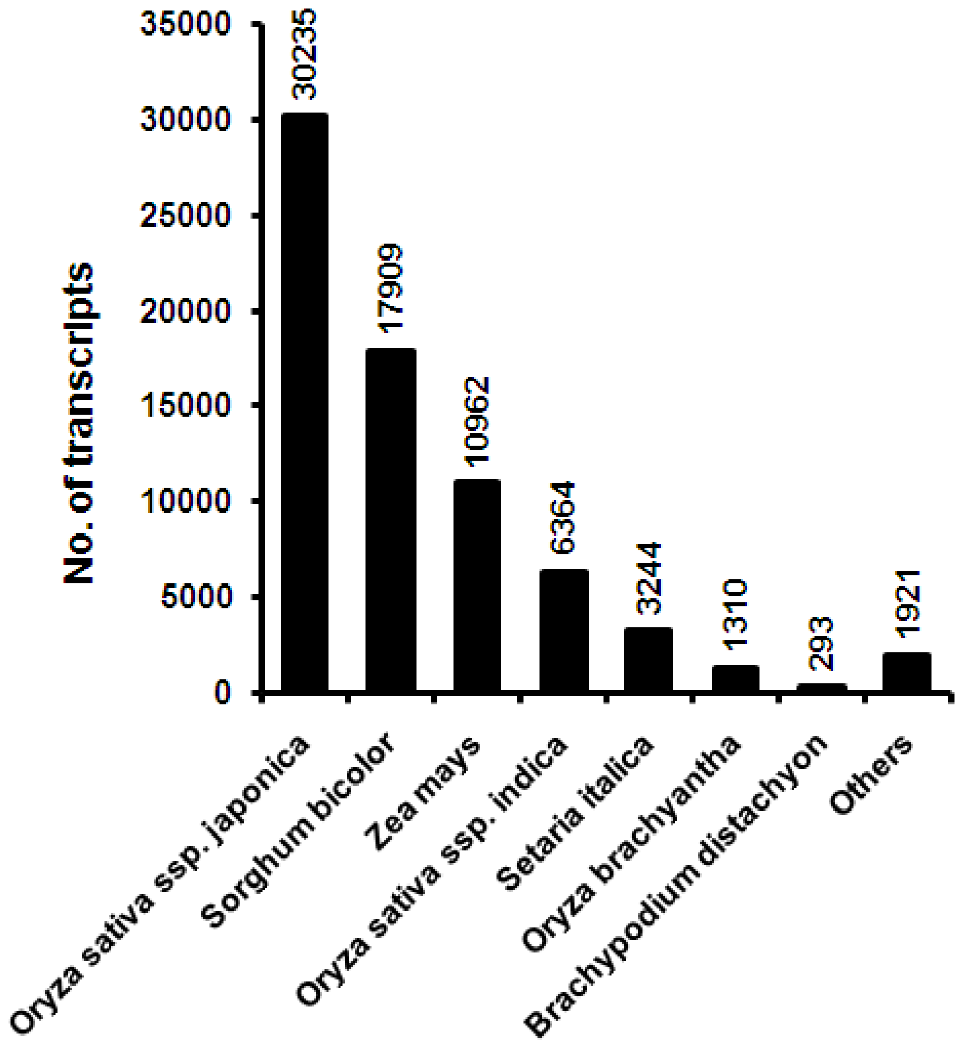

Figure S2 Top-hit species distribution of BLASTX matches for *C. flexuosus* transcripts.

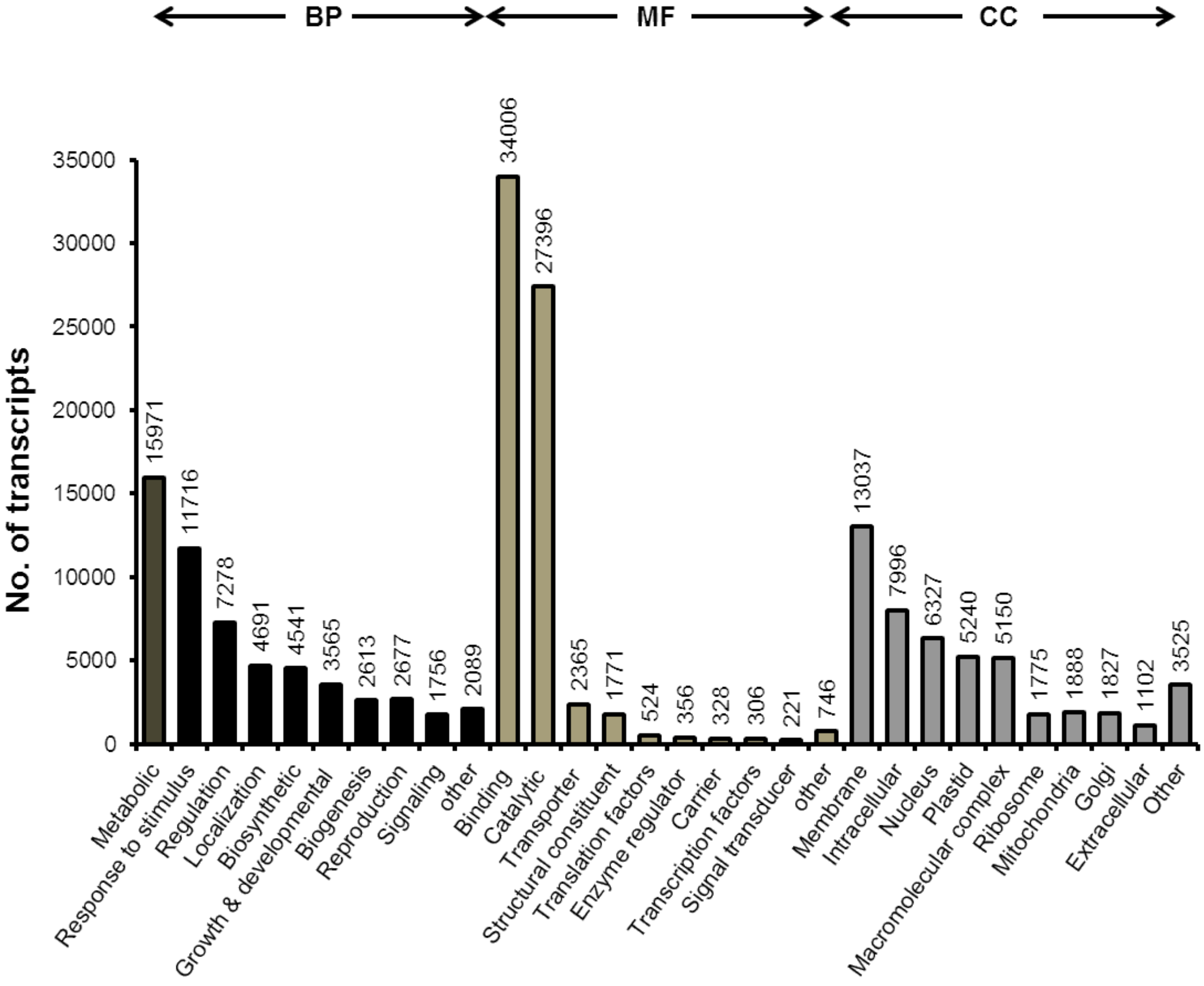

Figure S3 Gene ontology distribution of transcripts

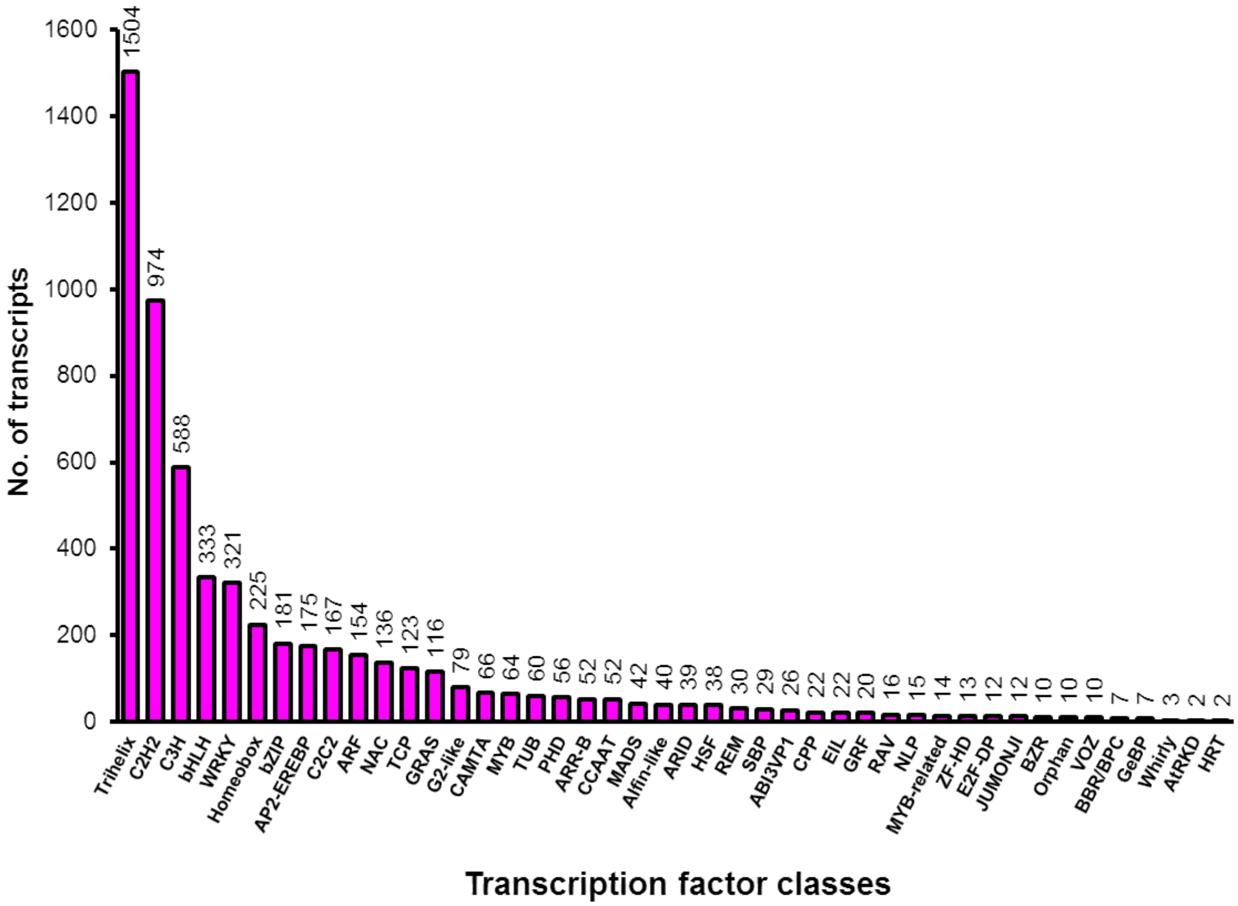

Figure S4 Distribution of transcripts into transcription factor classes based on AGRIS annotations. Transcription factors were identified using BLASTX search against AGRIS database (http://arabidopsis.med.ohio-state.edu).

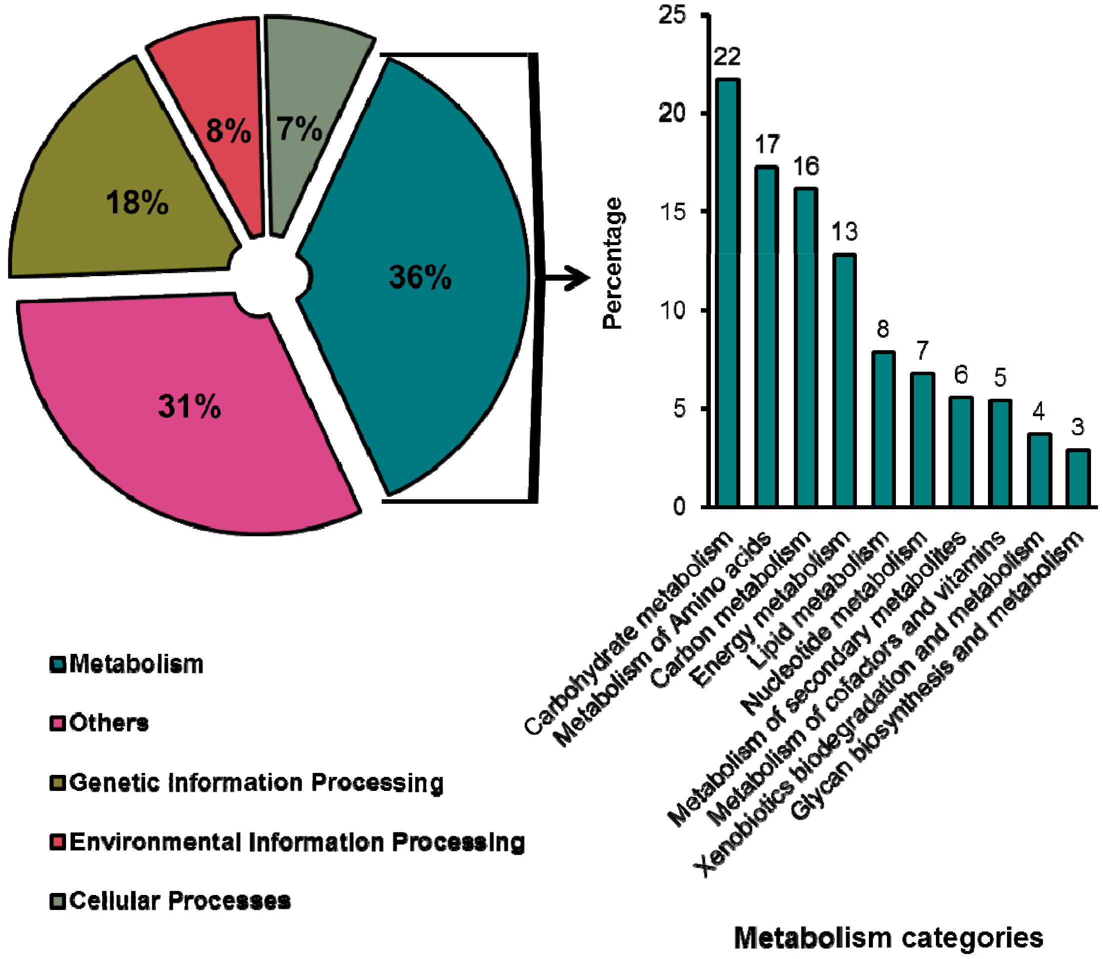

Figure S5 Percentage distribution of transcripts into KEGG orthologous categories. Right panel shows the percentage distribution of transcripts into sub-categories of metabolism.

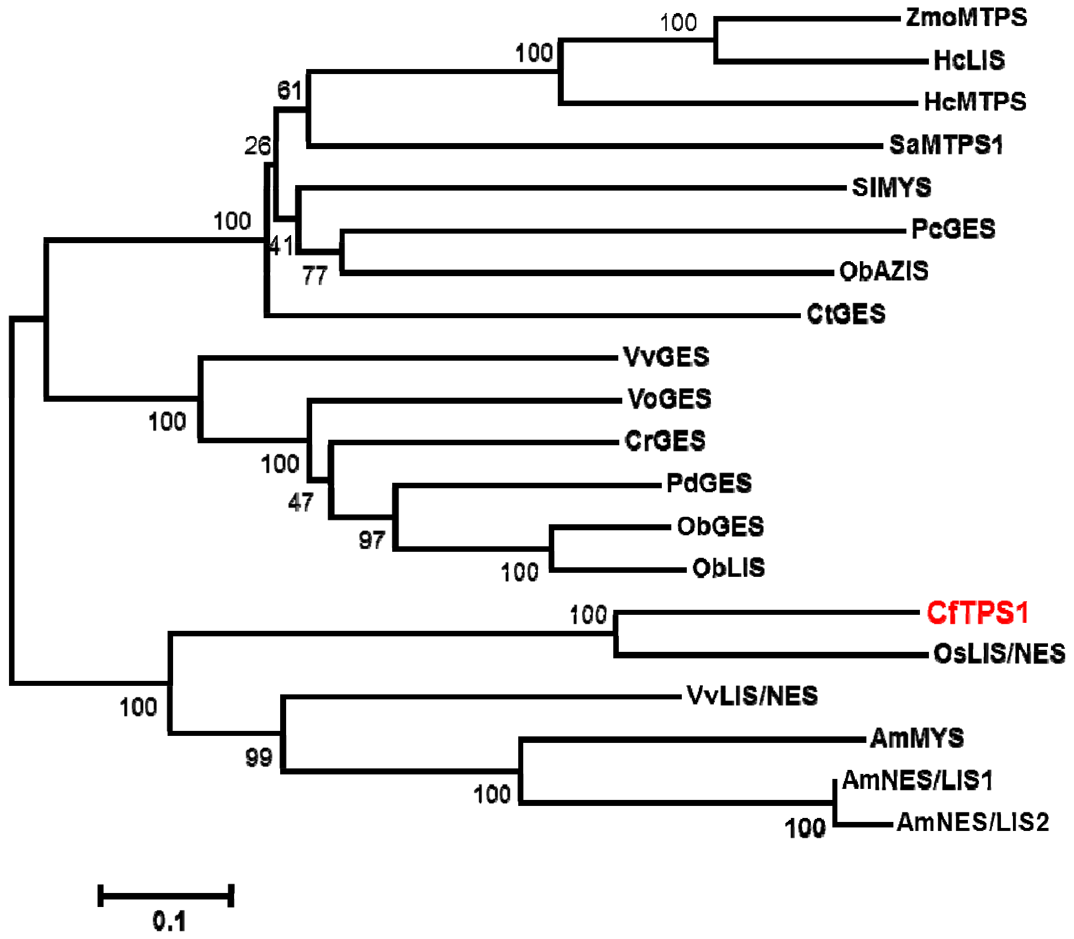

Figure S6 The phylogenetic relationship of *C. flexuosus* TPS1.

The evolutionary history was inferred using unrooted Neighbor-Joining (NJ) method with boot strap value of 1000. Evolutionary analyses were done using MEGA6. Abbreviation with accession numbers- *Antirrhinum majus* nerolidol/linalool synthase (AmNES/LIS1_ABR24417, AmNES/LIS2_ABR24418), *A. majus* myrcene synthase (AmMYS_AAO41727), *C. flexuosus* terpene synthase 1 (CfTPS1), *Catharanthus roseus* geraniol synthase (CrGES_), *Cinnamomum tenuipile* (CtGES_CAD29734), *Hedychium coronarium* (HcLIS_AER12203), *H. coronarium* (HcMTPS_AHJ57305), *Ocimum basilicum*α- zingiberene synthase (ObAZIS_Q5SBP4), *O. basilicum* geraniol synthase (ObGES_Q6USK1), *O.basilicum* linalool synthase (ObLIS_Q5SBP3), *Oryza sativa* linalool/nerolidol synthase (OsLIS/NES_ACF05530), *Perilla citriodora* geraniol synthase (PcGES_ABB30217), *Phyla dulcis* geraniol synthase (PdGES_ADK62524), *Santalum album* monoterpene synthase (SaMTPS1_B5A434), *Solanum lycopersicum* myrcene synthase (SlMYS_G1JUH1), *Valeriana officinalis* geraniol synthase (VoGES_AHE41084), *Vitis vinifera* geraniol synthase (VvGES_NP_001267920), *V. vinifera* linalool/nerolidol synthase (VvLIS/NES_AEY82696), *Zingiber montanum* monoterpene synthase (ZmoMTPS_AHI46572).

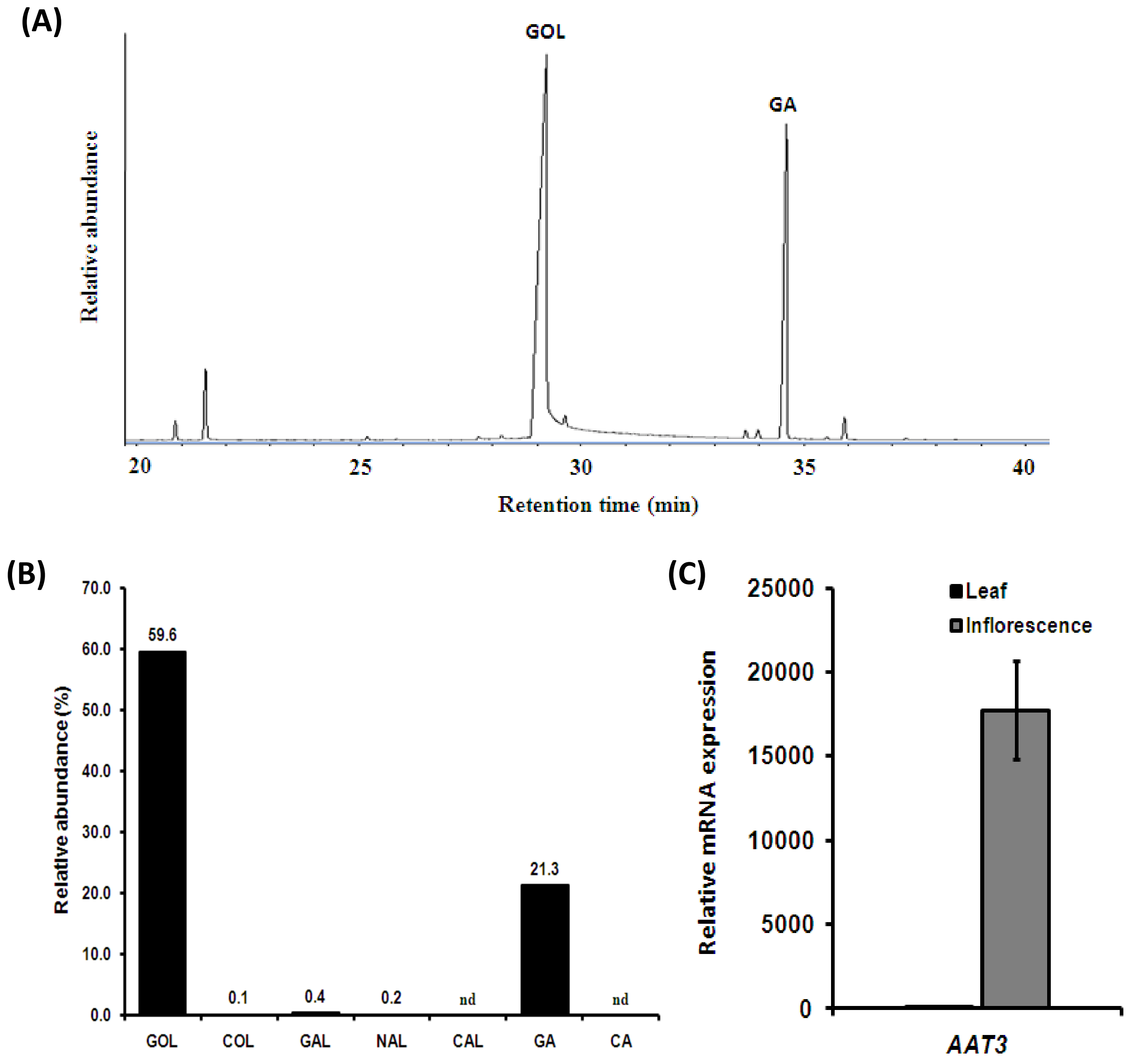

Figure S7 GC-MS profile (top) and relative quantification (bottom) of individual components in *C. martinii* inflorescence.

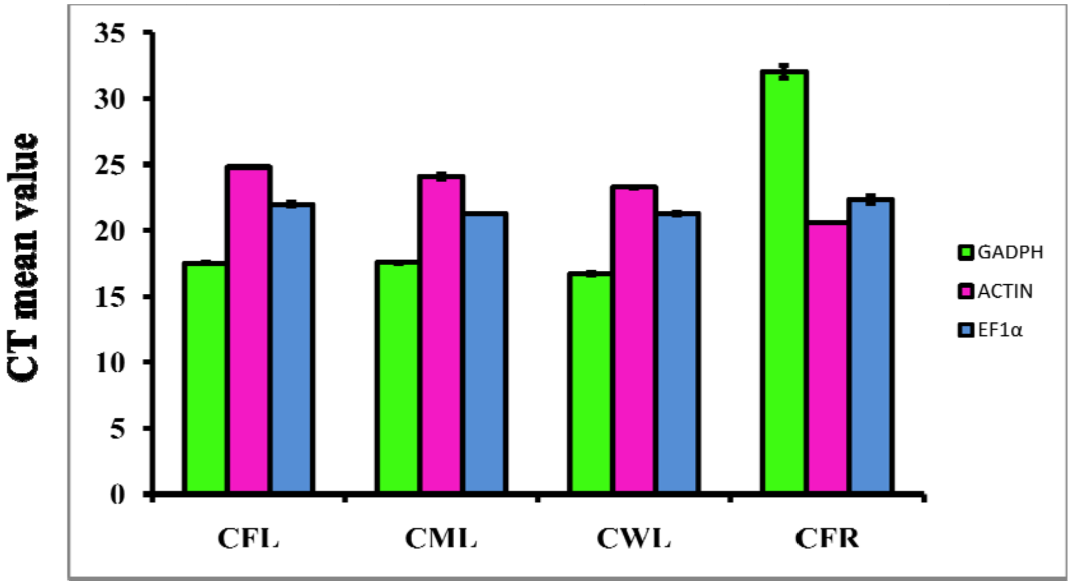

Figure S8A Expression profiling of reference genes in different species and tissues of *Cymbopogon* sp. Elongation factor 1α (*EF1α*), *actin* and glyceraldehydes 3- phospahate dehydrogenase (*GAPDH*) were used to check the stability across different *Cymbopogon* species and also in different tissues of *C. flexuosus*. CFL, *C. flexuosus* leaf; CML, *C. martini* leaf; CWL, *C. winterianus* leaf; and CFR, *C. flexuosus* root.

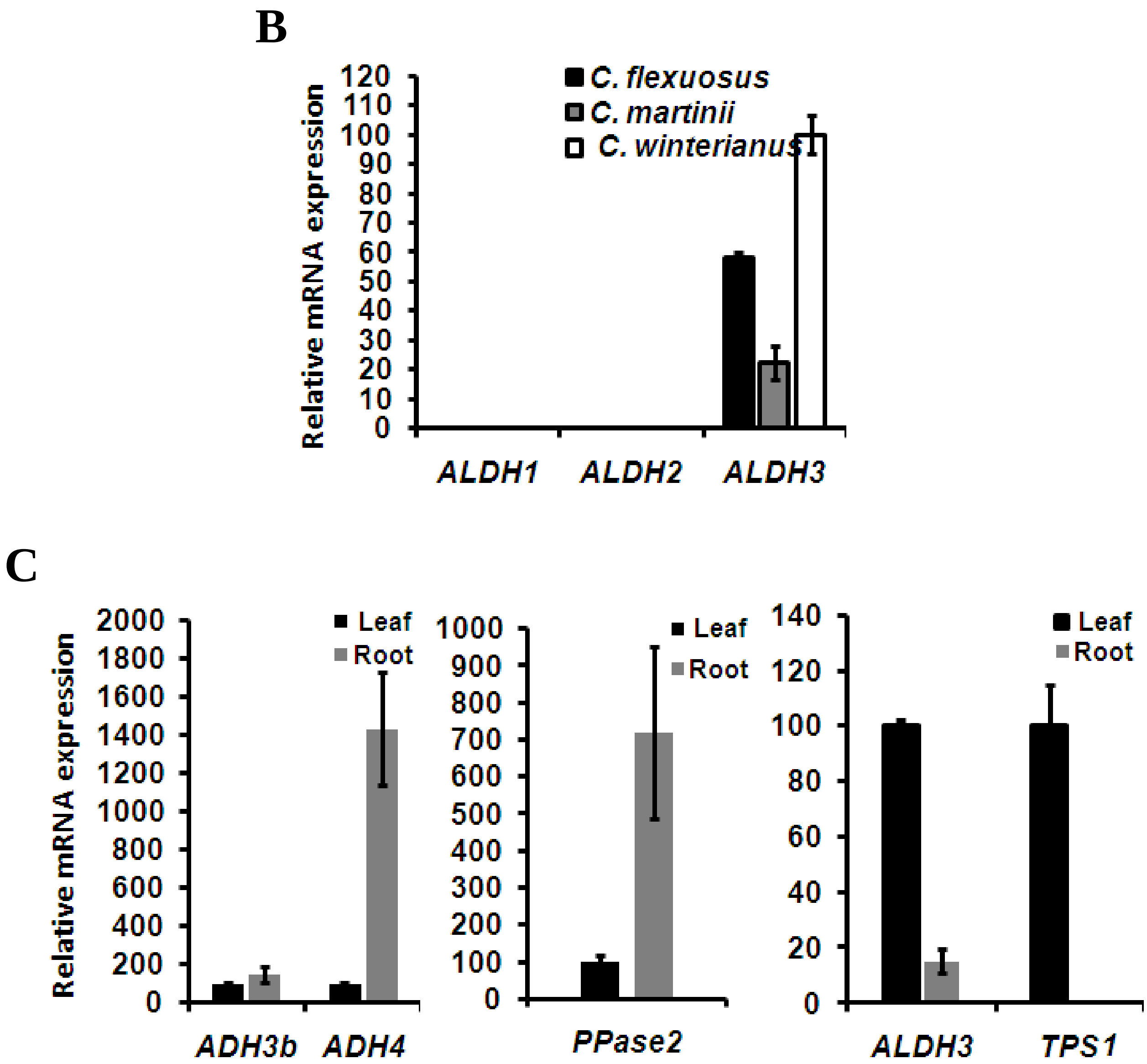

Figure S8B and C Relative expression analysis in *Cymbopogon*.

Comparative expression of *ALDH* candidates (b) and tissue specific expression of *TPS1*, *PPase1*, *ADH3b, ADH4* and *ALDH3* in *C. flexuosus* (c).

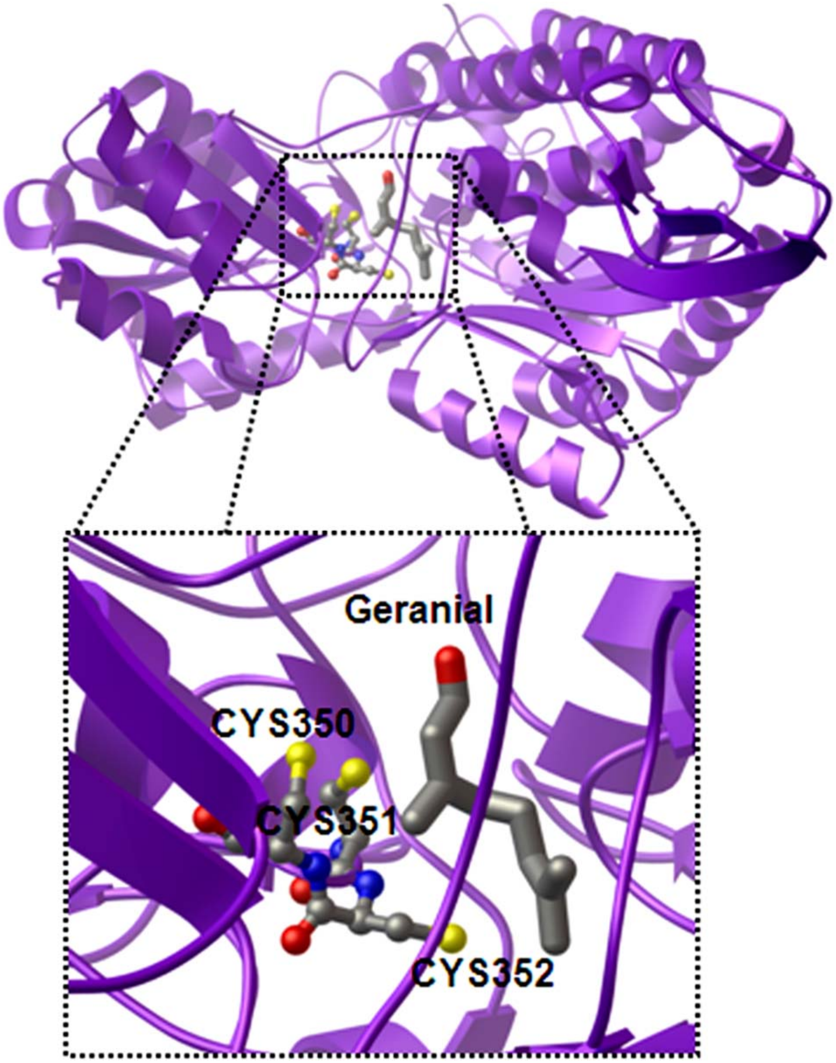

Figure S9 Three-dimensional ribbon model of the complete structure of CfALDH3. The homology model was built using the X-ray structures of *Bos taurus* ALDH (PDB ID: 1AG8). The substrate bound complexes were visualized by PMV software (http://mgltools.scripps.edu). The active site residues are represented as ball and stick, and substrates are shown as stick (coloured by atom type).

(A)

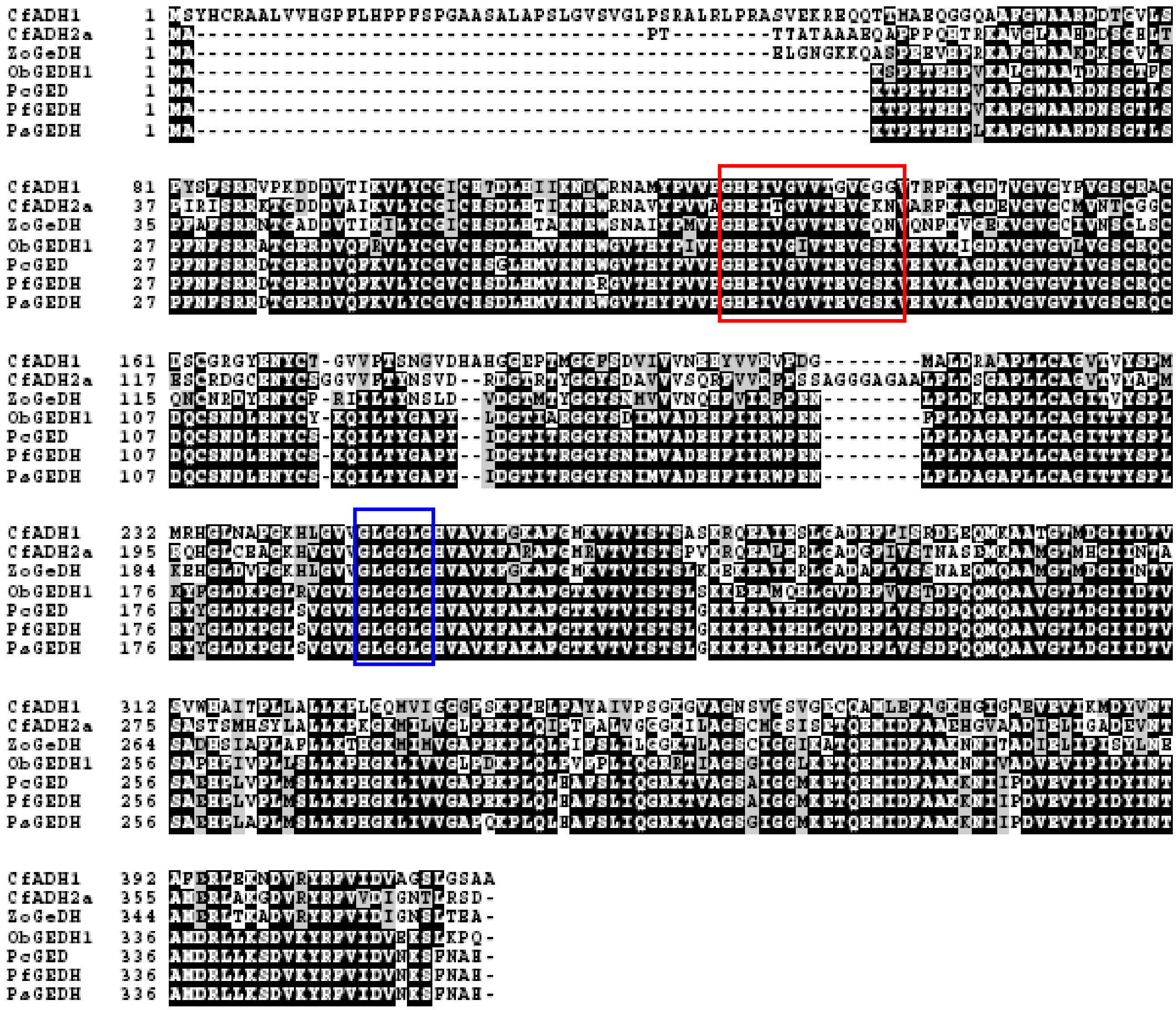

(B)

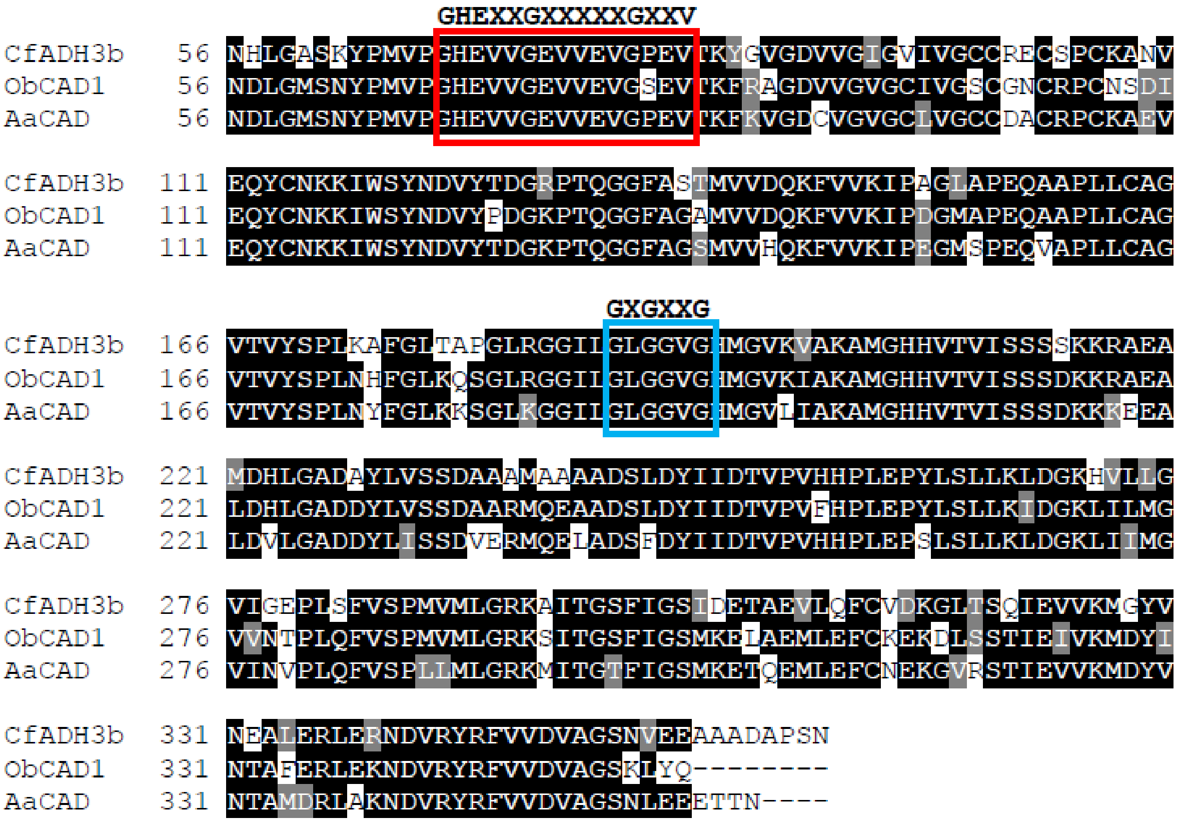
CfADH3b 1 MGSLASERKVVGWAARDATGHLSPYTYTLRNTGPEDVVVKVLYCGICHTDIHQAK
ObCAD1 1 MGSLEVERKTVGWAARDPSGVLSPYEYTLRNTGPQDVYVEVMCCGICHTDVHQIK
AaCAD 1 MGSMKEERKITGWAARDPSGVLAPYTYTLRNTGAEDVLIKVICCGICHTDPHQIK
GHEXXGXXXXXGXXV
CfADH3b 56 NHLGASKYPMVPGHEVVGEVVEVGPEVTKYGVGDVVGIGVIVGCCRECSPCKANV
ObCAD1 56 NDLGMSNYPMVPGHEVVGEVVEVGSEVTKFRAGDVVGVGCIVGSCGNCRPCNSDI
AaCAD 56 NDLGMSNYPMVPGHEVVGEVVEVGPEVTKFKVGDCVGVGCLVGCCDACRPCKAEV
CfADH3b 111 EQYCNKKIWSYNDVYTDGRPTQGGFASTMVVDQKFVVKIPAGLAPEQAAPLLCAG
ObCAD1 111 EQYCNKKIWSYNDVYFDGKPTQGGFAGAMVVDQKFVVKIPDGMAPEQAAPLLCAG
AaCAD 111 EQYCNKKIWSYNDVYTDGKPTQGGFAGSMVVHQKFVVKIPEGMSPEQVAPLLCAG
GXGXXG
CfADH3b 166 VTVYSPLKAFGLTAPGLRGGILGLGGVGHMGVKVAKAMGHHVTVISSSSKKRAEA
ObCAD1 166 VTVYSPLNHFGLKQSGLRGGILGLGGVGHMGVKIAKAMGHHVTVISSSDKKRAEA
AaCAD 166 VTVYSPLNYFGLKKSGLKGGILGLGGVGHMGVLIAKAMGHHVTVISSSDKKKEEA
CfADH3b 221 MDHLGADAYLVSSDAAAMAAAADSLDYIIDTVPVHHPLEPYLSLLKLDGKHVLLG
ObCAD1 221 LDHLGADDYLVSSDAARMQEAADSLDYIIDTVPVFHPLEPYLSLLKIDGKLILMG
AaCAD 221 LDVLGADDYLISSDVERMQELADSFDYIIDTVPVHHPLEPSLSLLKLDGKLIIMG
CfADH3b 276 VIGEPLSFVSPMVMLGRKAITGSFIGSIDETAEVLQFCVDKGLTSQIEVVKMGYV
ObCAD1 276 VVNTPLQFVSPMVMLGRKSITGSFIGSMKELAEMLEFCKEKDLSSTIEIVKMDYI
AaCAD 276 VINVPLQFVSPLLMLGRKMITGTFIGSMKETQEMLEFCNEKGVRSTIEVVKMDYV
CfADH3b 331 NEALERLERNDVRYRFVVDVAGSNVEEAAADAPSN
ObCAD1 331 NTAFERLEKNDVRYRFVVDVAGSKLYQ--------
AaCAD 331 NTAMDRLAKNDVRYRFVVDVAGSNLEEETTN----

(C)

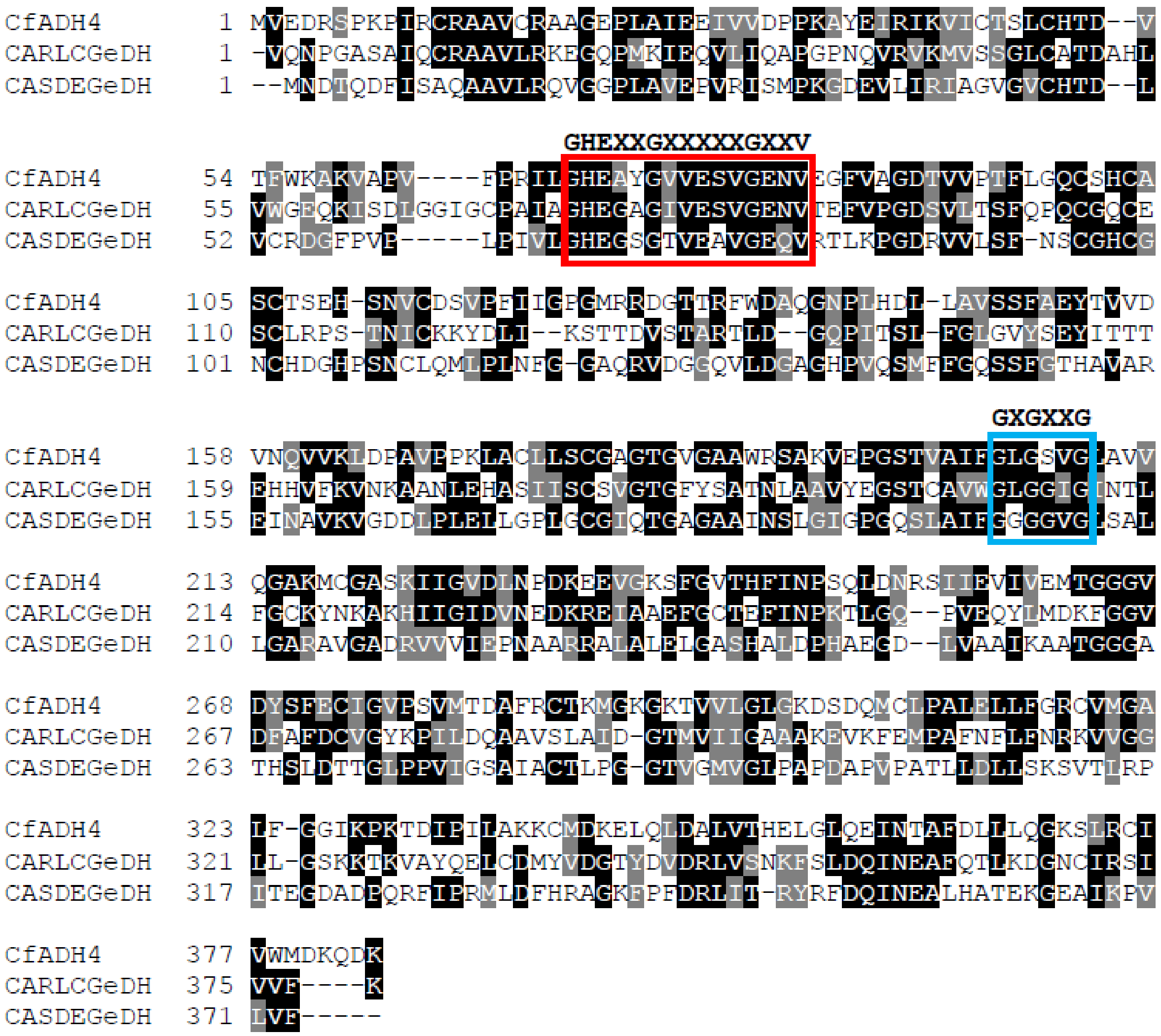

Figure S10 Multiple sequence alignment of CfADH1 and CfADH2a (a), CfADH3b (b), and CfADH4 (c) with the characterized GeDH and CAD. Abbreviation with accession numbers-CAD, Cinnamayl alcohol dehydrogenase; GeDH, Geraniol dehydrogenase. Species: Aa-*Artemisia annua* (ACB54931); Cd, *Castellaniellade fragrans* (B2N193); Cf, *Cymbopogon flexuosus*; Cl, *Carpoglyphus lactis* (HIZV38); *Ocimum basilicum* (ObCAD_AAX83108 and ObGeDH_AAX831C7); Pc, *Perilla citriodora* (AFY63473); Pf, *P. frutescens* (AFY63472); Ps, *P. setoyensis* (AFY63474) and Zo, *Zingiber officinale* (BAR42579). Alignment was built using MAFFT 7 and BOXSHADE 3.21. The catalytically active zinc binding motif and glycine reich phosphate binding loop are boxed in red and blue, respectively.

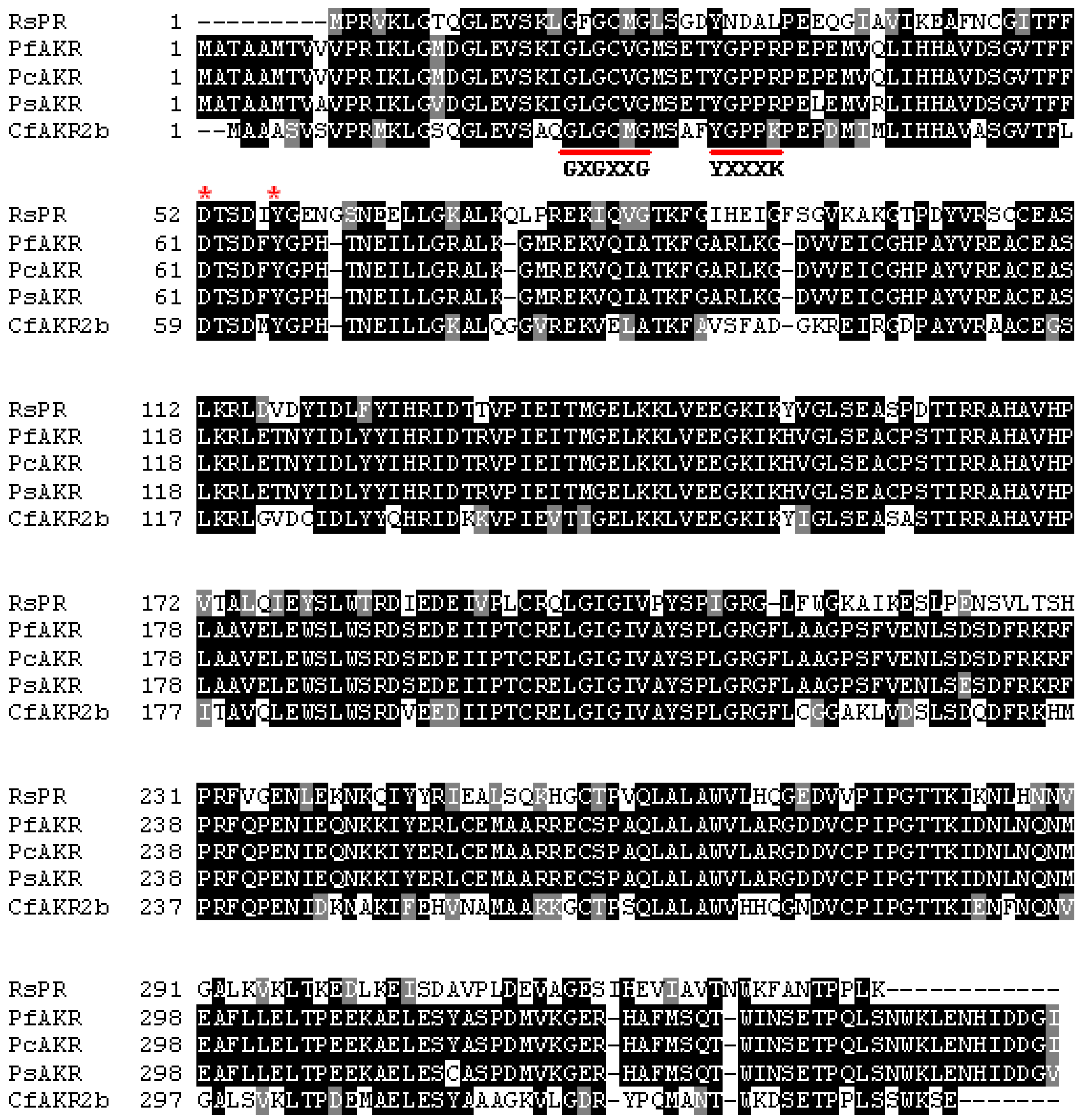

Figure S11 Multiple sequence alignment of CfAKR2b. Abbreviation- Bd, *Brachypodium distachyon* (XP_003575318); Ob, *Oryza brachyantha* (XP_006652179); Pc, *Perilla citriodora* (AFV99149); Pf, *Perilla frutescens* (AFV99148); Ps, *Perilla setoyensis* (AFV99150) and *R. serpentina* perakine reductase (3V0T_A). Alignment was built using MAFFT version 7 and BOXSHADE version 3.21. The catalytic residues are indicated by *. Cofactor binding motif (GXGXXC) and conserved residues are underlined in red.

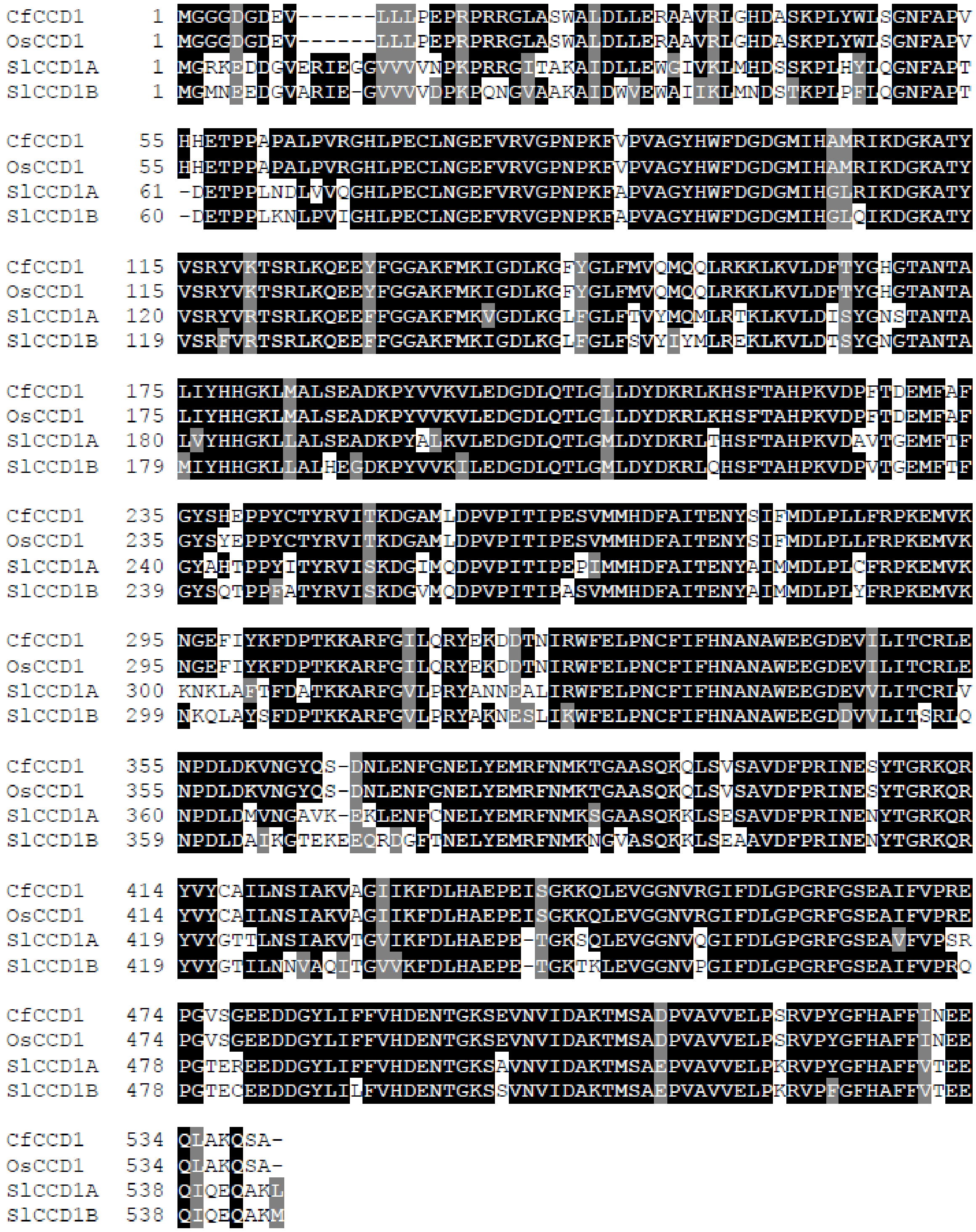

Figure S12 Multiple sequence alignment of CfCCD1 with characterized rice and tomato CCDs. Abbreviation- CCD, carotenoid cleavage dioxygenase. Species- Os, *Oryza sativa* (AK066766) and Sl, *Solanum lycopersicum* (AAT68187 and AAT68188). Alignment was built using MAFFT version 7 and BOXSHADE version 3.21.

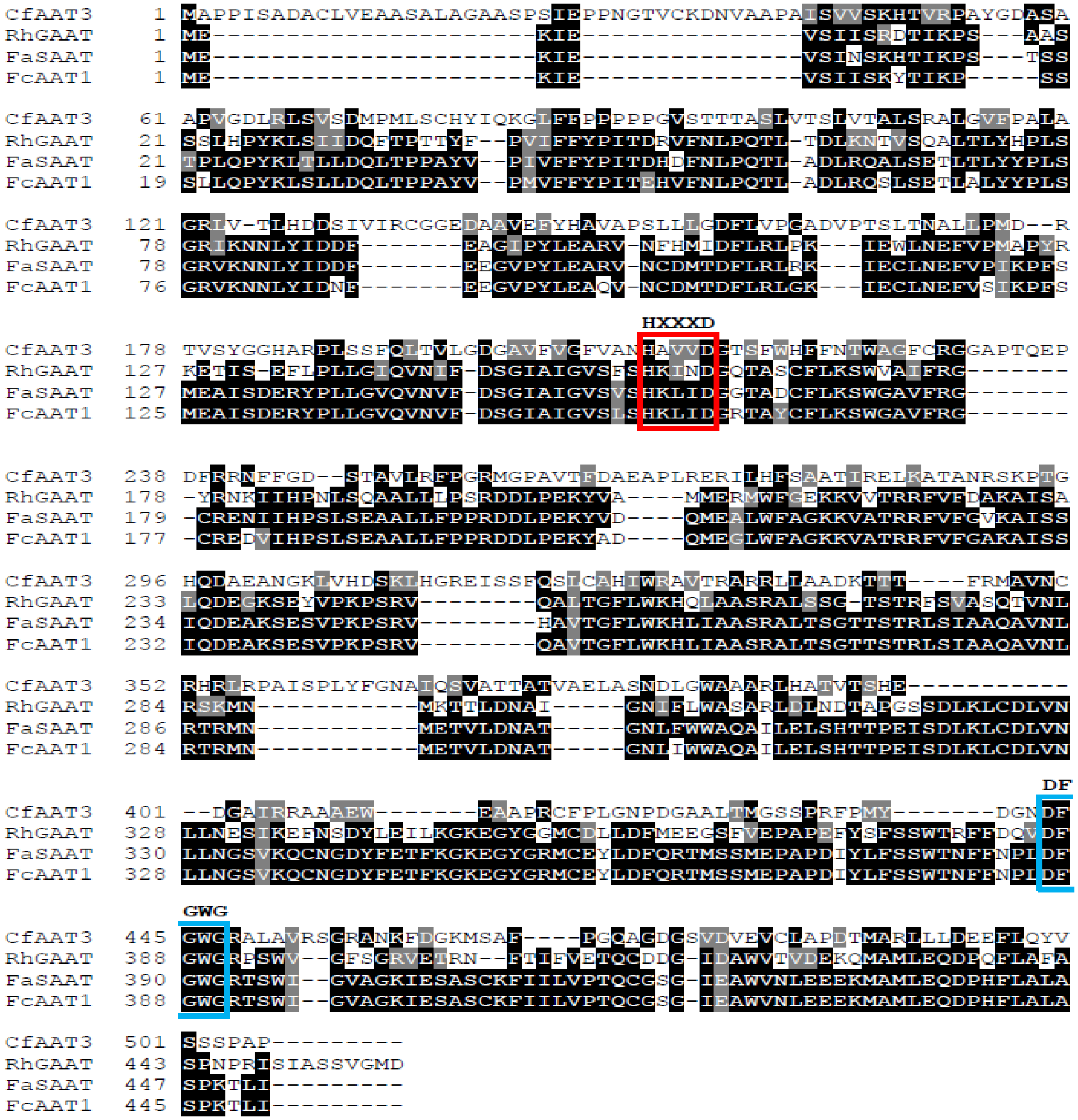

Figure S13 Sequence alignment of CfAAT3 with AATs from other plants. Abbreviation- FaAAT2, *Fragaria ananassa* alcohol acyltransferase 2 (AAG13130); FcAAT, *Fragaria chiloensis* alcohol acyltransferase (ACT822471); RhGAAT, *Rosa hybrida* acetyl CoA geraniol/citronellol acetyltransferase (AAW31948). Alignment was built using MAFFT version 7 and BOXSHADE version 3.21. The catalytic and structural motifs are boxed in red and blue, respectively.

```
CfALDH3     1 ---MARRAASSLISRCLLARAASSAPAGPSPSALRRPVPADGMRGLLPGVLQRFST----
ZmRF2B      1 MAATVRRAASSVLSRFLLTK--------PSPSPASAAGNNSALLGSGAAALHRFSTAPAS

CfALDH3    54 -AAAVEEPITPSVQVNYTKLLINGNFVDSASGKTFPTLDPRTGEVIAHVAEGDAEDINRA
ZmRF2B     53 AAAAAEEPIQPAVEVKHTQLLINGNFVDAASGKTFPTLDPRTGEVIARVAEGDSEDIDRA

CfALDH3   113 VAAARKAFDEGPWPKMTAYERSRILLRFADLIEKHNDELAALETWDNGKPYEQAAQIEVP
ZmRF2B    113 VAAARRAFDEGPWPRMTAYDRCRVLLRFADLIERHAEEVAALETWDNGKTLAQAAGAEVP

CfALDH3   173 MVARLMRYYAGWADKIHGLVVPADGPHHVQILHEPIGVAGQIIPWNFPLLMFAWKVGPAL
ZmRF2B    173 MVARCVRYYAGWADKIHGLVAPADGAHHVQVLHEPVGVAGQIIPWNFPLLMFAWKVGPAL

CfALDH3   233 ACGNTIVLKTAEQTPLSALYISKLLHEAGLPEGVVNVVSGYGPTAGAALASHMDVDKVAF
ZmRF2B    233 ACGNTVVLKTAEQTPLSALYVANLLHEAGLPEGVLNVVSGFGPTAGAALSSHMGVDKLAF

CfALDH3   293 TGSTDTGKIILELAAKSNLKTVTLELGGKSPFIIMGDADVDHAVELAHFALFFNQGQCCC
ZmRF2B    293 TGSTGTGQIVLELAARSNLKPVTLELGGKSPFIVMDDADVDQAVELAHQAVFFNQGQCCC

CfALDH3   353 AGSRTFVHERVYDEFVEKSKARALKRVVGDPFRKGVEQGPQIDDEQFNKILRYIRSGVDS
ZmRF2B    353 AGSRTFVHERVYDEFVEKSKARALKRVVGDPFRDGVEQGPQIDGEQFNKILRYVQSGVDS

CfALDH3   413 GANLVTGGDRLGDKGYYIQPTIFSDVQDGMKIAQEEIFGPVQSILKFKDLNEVIKRANAS
ZmRF2B    413 GATLVAGGDRVGDRGFYIQPTVFADAKDEMKIAREEIFGPVQTILKFSGVEEVIRRANAT

CfALDH3   473 QYGLAAGVFTNNLDTANTLARALRVGTVWTNCFDIFDAAIPFGGYKMSGHGREKGIDSLK
ZmRF2B    473 PYGLAAGVFTRSLDAANTLSRALRAGTVWVNCYDVFDATIPFGGYKMSGVGREKGIYALR

CfALDH3   533 NYLQVKAVVTPIKNAAWL
ZmRF2B    533 NYLQTKAVVTPIKNPAWL
```

Figure S14 Sequence alignment of CfALDH3 nearest characterized homologue. Abbreviation – Zm,*Zea mays* fertility restoration factor (RF)(AAL99613). Alignment was built using MAFFT version 7 and BOXSHADE version 3.21.

Protein sequences used in this study.

>CfTPS1
MSAAPVRIFSSSMEPLLLSSASPAATTAANNSRQGRRHRGDSIRPLSSSSSAVNTLLLRNDFDFQEGLKN
VLHQRQKSAREMMVTIDNLKRLCIDHYFEEEIESAMSSCMDLVHSNDLFDATLAFMLLREAGHDVSANDV
LRRFTDDSGEFKLPLSMDIRGLLSLHDMSHLDIGGEVLLYKAKEFSSKHLTSAIRYLEPSLAEYVRQSLD
HPYHRSLMQYKARHHLTYLQSLPIRDTVVEKLAVEEFQLNKLLHQQEVQEVNRWWMDLGLVQEIPVVRDQ
VLKWYMWSMTALQGYSFSRYRVEITKIIALVYVVDDIFDLVGTLEELSLFTEAVKVWNTAAADSLPSCMR
SCYMALYTITNEIADMAEKEHGLNPVNHLKKAWAVLFDGFLVEAKWLATDQVPTAEDYLRNGVITSGVPL
TLVHIFIMLGCDQSTEPLIDQMPSIISCPAKILRLWDDMGSAEDEAQEGLDGSYRDFYLIENPICGPSDA
EAHMRSLIAREWEELNRECLCKRSFSSNFTQTCLNVTRMISVMYSYNKEQRLLVLEDYARMLIL

>CfPPase1
MATAATASATAATRFTLLAGAGLRSRISIIRRRPTTAVRFQRQQRGLTTTALLKTAELLPKTQGQPETLD
YRVFLVDGGGRKVSPWHDVPLRAGDGVFHFVVEIPKESSAKMEVATDEPFTPIKQDTKKGNLRYYPYNIN
WNYGLLPQTWEDPTSANSEVEGAFGDNDPVDVVEIGERRANVGEVLKVKPLAALAMIDEGELDWKIVAIS
LDDPKASLVNDVDDVEKHFPGTLTAIRDWFRDYKIPDGKPANKFGLGNKPASKEYALKVIEETNESWEKL
VKRNIPAGELSLA

>CfPPase2
MSEEDKTAASAEQPKRAPKLNERILSSLSRRSVAAHPWHDLEIGPGAPAVFNVVVEITKGSKVKYELDKK
TGLIKVDRVLYSSVVYPHNYGFIPRTLCEDNDPMDVLVLMQEPVIPGSFLRARAIGLMPMIDQGEKDDKI
IAVCADDPEYRHYNDISELSPHRLQEIKRFFEDYKKNENKEVAVDAFLPATTAREAIQYSMDLYAQYILQ
SLRQ

>CfAE1
MGCSWALAALVLGFLVVAVHGSEPWLNQTQVYSTNANSGSNGVFVGITLIQSAAAKGAVCLDGSLPGYHL
HRGFGSGANSWLVNLEGGGWCNDVKSCVFRKSSRRGSSNHMESQLQFTGIMSNRPEENPDFYNWNRVKVR
YCDGGSFTGDGADASAGLYFRGQRIWQAAMDDLMAQGMRYANQALLSGCSAGGVSTILHCDEFRGLFSGS
TNVKCLADAGMFLDFVDVSGQREMRDFFNGIVRLQGSGRSLPRSCTSRMDKTSCFFPQNVVPNIQTPTFI
LNTAYDVWQLQQSVAPKRADPQGLWRGCRMNHASCNSNQLQFLQGFRNQMLDAVRGFSGARQNGLFINSC
FAHCQSERQDTWYAGDSPRLGNKRIAEAVGDWFFDRADAKYTDCAYPCDGTCHHLTFRGDY

>CfADH1
MSYHCRAALVVHGPFLHPPFSPGAASALAPSLGVSVGLPSRALRLPRASVEKREQQTTMAEQGGQAAFGW
AARDDTGVLSPYSFSRRVPKDDDVTIKVLYCGICHTDLHIIKNDWRNAMYPVVPGHEIVGVVTGVGGGVT
RFKAGDTVGVGYFVGSCRACDSCGRGYENYCTGVVPTSNGVDHAHGGEPTMGGFSDVIVVNEHYVVRVPD
GMALDRAAPLLCAGVTVYSPMMRHGLNAPGKHLGVVGLGGLGHVAVKFGKAFGMKVTVISTSASKRQEAI
ESLGADEFLISRDPEQMKAATGTMDGIIDTVSVWHAITPLLALLKPLGQMVIGGGPSKPLELPAYAIVPS
GKGVAGNSVGSVGECQAMLEFAGKHGIGAEVEVIKMDYVNTAFERLEKNDVRYRFVIDVAGSLGSAA

>CfADH2a
MAPTTTATAAAEQAPPPQHTRKAVGLAAHDDSGHLTPIRISRRKTGDDDVAIKVLYCGICHSDLHTIKNE
WRNAVYPVVAGHEITGVVTEVGKNVARFKAGDEVGVGCMVNTCGGCESCRDGCENYCSGGVVFTYNSVDR
DGTRTYGGYSDAVVVSQRFVVRFPSSAGGGAGAALPLDSGAPLLCAGVTVYAPMRQHGLCEAGKHVGVVG
LGGLGHVAVKFARAFGMRVTVISTSPVKRQEALERLGADGFIVSTNASEMKAAMGTMHGIINTASASTSM
HSYLALLKPKGKMILVGLPEKPLQIPTFALVGGGKILAGSCMGSISETQEMIDFAAEHGVAADIELIGAD
EVNTAMERLAKGDVRYRFVVDIGNTLRSD

>CfADH2b
MAPTTTATAAAEQAPPPQHTRKAVGLAAHDDSGHLTPIRISRRKTGDDDVAIKVLYCGICHSDLHTIKNE
WRNAVYPVVAGHEITGVVTEVGKNVARFKAGDEVGVGCMVNTCGGCESCRDGCENYCSGGVVFTYNSVDR
DGTRTYGGYSDAVVVSQRFVVRFPSSAGGGAGAALPLDSGAPLLCAGVTVYAPMRQHGLCEAGKHVGVVG
LGGLGHVAVKFAKAFGMRVTVISTSPGKRREALEHLGADEFLVSRDAGQMAAAAATMDGILNTVSAWHPV

APLFALMKPMAQMVFVGAPTRPLELPAYAIVPGGKGITGNCVGGIRDCQAMLDFAGEHGITAEVEVIKMD
YVNTAMERLEKNDVRYRFVIDVAGSSLAGSGDAKI

>CfADH3a
MGSLASERKVVGWAARDATGHLSPYTYTLRNTGPEDVVVKVLYCGICHTDIHQAKNHLGASKYPMVPGHE
VVGEVVEVGPEVTKYGVGDVVGIGVIVGCCRECSPCKANVEQYCNKKIWSYNDVYTDGRPTQGGFASTMV
VDQKFVVKIPAGLAPEQAAPLLCAGVTVYSPLKAFGLTAPGLRGGILGLGGVGHMGVKVAKAMGHHVTVI
SSSSKKRAEAMDHLGADAYLVSSDAAAMAAAADSLDYIIDTVPVHHPLEPYLSLLKLDGKHVLLGVIGEP
LSFVSPMVMLGRKAITGSFIGSIDETAEVLQFCVDKGLTSQIEVVKMGYVNEALERLERNDVRYRFVVDV
AGSNIDDADAPPA

>CfADH3b
MGSLASERKVVGWAARDATGHLSPYTYTLRNTGPEDVVVKVLYCGICHTDIHQAKNHLGASKYPMVPGHE
VVGEVVEVGPEVTKYGVGDVVGIGVIVGCCRECSPCKANVEQYCNKKIWSYNDVYTDGRPTQGGFASTMV
VDQKFVVKIPAGLAPEQAAPLLCAGVTVYSPLKAFGLTAPGLRGGILGLGGVGHMGVKVAKAMGHHVTVI
SSSSKKRAEAMDHLGADAYLVSSDAAAMAAAADSLDYIIDTVPVHHPLEPYLSLLKLDGKHVLLGVIGEP
LSFVSPMVMLGRKAITGSFIGSIDETAEVLQFCVDKGLTSQIEVVKMGYVNEALERLERNDVRYRFVVDV
AGSNVEEAAADAPSN

>**CfADH3c**
MGSLAAEKTVTGWAARDASGHLTPYNYTLRKTGPEDVVVKVLYCGICHTDIHQAKNHLGASKYPMVPGHE
VVGEVVEVGPEVTKYSAGDVVGVGVIVGCCRECHPCKANVEQYCNKRIWSYNDVYTDGRPTQGGFASAMV
VDQKFVVKIPAGLAPEQAAPLLCAGVTVYSPLKAFGLTAPGLRGGILGLGGVGHMGVKVAKAMGHHVTVI
SSSSKKRAEAMDHLGADAYLVSSDAAAMAAAADSLDYIIDTVPVHHPLEPYLSLLKLDGKHVLLGVIGEP
LSFVSPMVMLGRKAITGSFIGSIDETAEVLQFCVDKGLTSQIEVVKMGYVNEALERLERNDVRYRFVVDV
AGSNIDDADAPPA

>**CfADH3d**
MGSLAAEKTVTGWAARDASGHLTPYNYTLRKTGPEDVVVKVLYCGICHTDIHQAKNHLGASKYPMVPGHE
VVGEVVEVGPEVTKYSAGDVVGVGVIVGCCRECHPCKANVEQYCNKRIWSYNDVYTDGRPTQGGFASAMV
VDQKFVVKIPAGLAPEQAAPLLCAGLTVYSPLKHFGLMSPGLRGGVLGLGGVGHMGVKVAKSMGHHVTVI
SSSARKRGEAMDDLGADAYLVSSDAAAMAAAGDSLDYIIDTVPVHHPLEPYLALLKLDGKLILMGVINQP
LSFISPMVMLGRKAITGSFIGSMAETEEVLNFCVDKGLTSQIEVVKMGYVNEALERLERNDVRYRFVVDV
AGSNIDDADAPPA

>**CfADH3e**
MGSLAAEKTVTGWAARDASGHLTPYNYTLRKTGPEDVVVKVLYCGICHTDIHQAKNHLGASKYPMVPGHE
VVGEVVEVGPEVTKYSAGDVVGVGVIVGCCRECHPCKANVEQYCNKRIWSYNDVYTDGRPTQGGFASAMV
VDQKFVVKIPAGLAPEQAAPLLCAGVTVYSPLKAFGLTAPGLRGGILGLGGVGHMGVKVAKAMGHHVTVI
SSSSKKRAEAMDHLGADAYLVSSDAAAMAAAADSLDYIIDTVPVHHPLEPYLSLLKLDGKHVLLGVIGEP
LSFVSPMVMLGRKAITGSFIGSIDETAEVLQFCVDKGLTSQIEVVKMGYVNEALERLERNDVRYRFVVDV
AGSNVEEAAADAPSN

>**CfADH4**
MVEDRSPKPIRCRAAVCRAAGEPLAIEEIVVDPPKAYEIRIKVICTSLCHTDVTFWKAKVAPVFPRILGH
EAYGVVESVGENVEGFVAGDTVVPTFLGQCSHCASCTSEHSNVCDSVPFIIGPGMRRDGTTRFWDAQGNP
LHDLLAVSSFAEYTVVDVNQVVKLDPAVPPKLACLLSCGAGTGVGAAWRSAKVEPGSTVAIFGLGSVGLA
VVQGAKMCGASKIIGVDLNPDKEEVGKSFGVTHFINPSQLDNRSIIEVIVEMTGGGVDYSFECIGVPSVM
TDAFRCTKMGKGKTVVLGLGKDSDQMCLPALELLFGRCVMGALFGGIKPKTDIPILAKKCMDKELQLDAL
VTHELGLQEINTAFDLLLQGKSLRCIVWMDKQDK

>**CfAKR1**
MAAAAATAPAAAVVRRMKLGSQGMEVSAQGLGCMGMSAVYGERKPEADMVALVRHAVAAGVTFLDTSDVY

GPHTNEVLVGKAVAAAAATEEEVQVQVATKFGITPAWEVRGDPAYVRAACEGSLRRLGVGCIDLYYQHRI
DSTVPVEITMGELKKLVEEGKIKYIGLSEASASTIRRAHVVHPITAVQIEWSLWSRDVEEDIVPTCRELG
IGIVAYSPLGRGFFSSGAKLVDELPDDDFRKSLPRFQPENLEKNAAIFEKVNAMAARKGCTSSQLALAWV
HHQGSDVCPIPGTTKIHNFDQNVGALSVKLTPDEMSELESYASADVVQGDRYHGTFLNTWKNSETPPLSS
WRSGN

>**CfAKR2a**
MAAASVSVPRMKLGSQGLEVSAQGLGCMGMSAFYGPPKPEPDMIMLIHHAVASGVTFLDTSDMYGPHTNE
ILLGKALQGGVREKVELATKFAVSFADGKREIRGDPAYVRAACEGSLKRLGVDCIDLYYQHRIDKKVPIE
VTIGELKKLVEEGKIKYIGLSEASASTIRRAHAVHPITAVQLEWSLWSRDVEEDIIPTCRELGIGIVAYS
PLGRGFLCGGAKLVDSLSDQDFRKHMPRFQPENIDKNAKIFEHVNAMAAKKGCTPSQLALAWVHHQGNDV
CPIPGTTKIENFNQNVGALSVKLTPDEMVELESYTAAGEVSGDRYAGITNTWKDSETPPLSSWKYE

>**CfAKR2b**
MAAASVSVPRMKLGSQGLEVSAQGLGCMGMSAFYGPPKPEPDMIMLIHHAVASGVTFLDTSDMYGPHTNE
ILLGKALQGGVREKVELATKFAVSFADGKREIRGDPAYVRAACEGSLKRLGVDCIDLYYQHRIDKKVPIE
VTIGELKKLVEEGKIKYIGLSEASASTIRRAHAVHPITAVQLEWSLWSRDVEEDIIPTCRELGIGIVAYS
PLGRGFLCGGAKLVDSLSDQDFRKHMPRFQPENIDKNAKIFEHVNAMAAKKGCTPSQLALAWVHHQGNDV
CPIPGTTKIENFNQNVGALSVKLTPDEMAELESYAAAGKVLGDRYPQMANTWKDSETPPLSSWKSE

>**CfCCD1**
MGGGDGDEVLLLPEPRPRRGLASWALDLLERAAVRLGHDASKPLYWLSGNFAPVHHETPPAPALPVRGHL
PECLNGEFVRVGPNPKFVPVAGYHWFDGDGMIHAMRIKDGKATYVSRYVKTSRLKQEEYFGGAKFMKIGD
LKGFYGLFMVQMQQLRKKLKVLDFTYGHGTANTALIYHHGKLMALSEADKPYVVKVLEDGDLQTLGLLDY
DKRLKHSFTAHPKVDPFTDEMFAFGYSHEPPYCTYRVITKDGAMLDPVPITIPESVMMHDFAITENYSIF
MDLPLLFRPKEMVKNGEFIYKFDPTKKARFGILQRYEKDDTNIRWFELPNCFIFHNANAWEEGDEVILIT
CRLENPDLDKVNGYQSDNLENFGNELYEMRFNMKTGAASQKQLSVSAVDFPRINESYTGRKQRYVYCAIL
NSIAKVAGIIKFDLHAEPEISGKKQLEVGGNVRGIFDLGPGRFGSEAIFVPREPGVSGEEDDGYLIFFVH
DENTGKSEVNVIDAKTMSADPVAVVELPSRVPYGFHAFFINEEQLAKQSA

>**CfAAT1**
MAITVRRSTMVRPAWETPRVRLWNSNLDLVVPRFHTPSVYFYRRGPEGGGAPEGFFDGERMRRALAEALV
PFYPMAGRLARDEDGRVEIDCNGEGVLFVEADAPDASVDDYGDFAPTMELKRLIPAVDYTDDISSFSLLV
LQVTYFKCGGVSLGVGMQHHVADGMSGLHFINSWSDLCRGTQIAIMPFIDRTLLRARDPPTPSYPHVEYQ
PAPAMLSSVPQSVTANKTTPPPTAVDIFKLTRSDLGRLRSQLPSGEGAPRFSTYAVLAAHVWRCVSLARG
LPSEQPTKLYCATDGRQRLQPPLPEGYFGNVIFTATPLAEAGKVTSGLADGAAVIQEALDRMNDSYCRSA
LDYLELQPDLSALVRGAHTFRCPNLGLTSWVRLPIHDADFGWGRPVFMGPGGIAYEGLAFVLPSANKDGS
LSIAISLQAEHMEKFRKLIFEV

>**CfAAT2**
MGIFTVTKLSEGPVRPSADTPSVTLPLAWVDRYPTHRGLVESTHIYCSDAAAMNTLLPPAPAAGGAEVDG
ALAAPKKEVVATKTMKSPAAVVRGALADALVHYYPFAGRIVEDVPGRPAVLCSAEGVYFVEAAANCTLAD
VNFLERPLLLAKEQLVPYPTPELWPVEPHNSLAMIQVTSFTCGGFVVGLRTNHAVADGTGAAQFLNAVGD
LARGLPEPRVKPVWGRDRFPDPDIKPGPLPELPVLALEYIAFDFPTAYLSKLKAQYAASTGGKICSGFDI
VIAKLWQCRTRAIDAGGTSDVDVRLCFFASVRHVLKLEPGYYGNAIFPVKVCAPAEKVAGSSVIELVGMV
REAKRRVAEECLAWAEGRTGGVDPFQMTFNYESVYVSDWSKLGFADVDYGYGAPMSAGPLVNCDLIASVI
VMRAPAPLAGTRLLASCVTKEHADDFARRMREDLLV

>**CfAAT3**
MAPPISADACLVEAASALAGAASPSIEPPNGTVCKDNVAAPAISVVSKHTVRPAYGDASAAPVGDLRLSV
SDMPMLSCHYIQKGLFFPPPPPGVSTTTASLVTSLVTALSRALGVFPALAGRLVTLHDDSIVIRCGGEDA
AVEFYHAVAPSLLLGDFLVPGADVPTSLTNALLPMDRTVSYGGHARPLSSFQLTVLGDGAVFVGFVANHA

VVDGTSFWHFFNTWAGFCRGGAPTQEPDFRRNFFGDSTAVLRFPGRMGPAVTFDAEAPLRERILHFSAAT
IRELKATANRSKPTGHQDAEANGKLVHDSKLHGREISSFQSLCAHIWRAVTRARRLLAADKTTTFRMAVN
CRHRLRPAISPLYFGNAIQSVATTATVAELASNDLGWAAARLHATVTSHEDGAIRRAAAEWEAAPRCFPL
GNPDGAALTMGSSPRFPMYDGNDFGWGRALAVRSGRANKFDGKMSAFPGQAGDGSVDVEVCLAPDTMARL
LLDEEFLQYVSSSPAP

>**CfALDH1**
MAAAAARRGSSLLSRCLLSRPAAAASPAVPSALRRADGTQGLLPGILQRFSTAAVAEEPISPPVQVNYTQ
LLIDGKFVDSASGKTFPTLDPRTGELIAHVAEGDAEDINRAVHAARKAFDEGPWPKMTAYERSRILLRFA
DLIEKHNDEIAALETWDNGKPYAQAANIEVPMVARLMRYYAGWADKIHGLVVPADGPHHVQVLHEPIGVA
GQIIPWNFPLLMFAWKVGPALACGNTVVLKTAEQTPLSALFASKLLHEAGLPDGVVNVVSGFGPTAGAAL
ASHMDVDKIAFTGSTDTGKVVLELAARSNLKSVTLELGGKSPFIIMDDADVDHAVELAHFALFFNQGQCC
CAGSRTFVHERIYDEFVEKAKARALKRVVGDPFKNGVEQGPQIDDEQFNKILRYIKYGVDSGANLVTGGD
RLGDKGYYIQPTIFSDVQDNMRIAQEEIFGPVQSILKFNDLNEVIKRANASQYGLAAGVFTNNLNTANTL
TRALRVGTVWVNCFDVFDAAIPFGGYKQSGIGREKGIDSLKNYLQVKAVVTPIKNAAWL

>**CfALDH2**
MAARRAASSLLSRGLIARPSAASSTGDSAILGAGSARGFLPGSLHRFSAAPAAAATAAATEEPIQPPVDV
KYTKLLINGNFVDAASGKTFATVDPRTGDVIARVAEGDAEDVNRAVAAARRAFDEGPWPRMTAYERCRVL
LRFADLIEQHADEIAALETWDGGKTLEQTTGTEVPMVARYMRYYGGWADKIHGLVVPADGPHHVQVLHEP
IGVAGQIIPWNFPLLMFAWKVGPALACGNAVVLKTAEQTPLSALFVASLLHEAGLPDGVLNVVSGFGPTA
GAALSSHMGVDKLAFTGSTGTGKIVLELAARSNLKPVTLELGGKSPFIVMDDADVDQAVELAHRALFFNQ
GQCCCAGSRTFVHERVYDEFVEKARARALQRVVGDPFRTGVEQGPQIDGEQFKKILQYVKSGVDSGATLV
AGGDRAGSRGFYIQPTVFADVEDEMKIAQEEIFGPVQSILKFSTVEEVVRRANATPYGLAAGVFTQRLDA
ANTLARALRVGTVWVNTYDVFDAAVPFGGYKMSGVGREKGVYSLRNYLQTKAVVTPIKDAAWL

>**CfALDH3**
MARRAASSLISRCLLARAASSAPAGPSPSALRRPVPADGMRGLLPGVLQRFSTAAAVEEPITPSVQVNYT
KLLINGNFVDSASGKTFPTLDPRTGEVIAHVAEGDAEDINRAVAAARKAFDEGPWPKMTAYERSRILLRF
ADLIEKHNDELAALETWDNGKPYEQAAQIEVPMVARLMRYYAGWADKIHGLVVPADGPHHVQILHEPIGV
AGQIIPWNFPLLMFAWKVGPALACGNTLVLKTAEQTPLSALYISKLLHEAGLPEGVVNVVSGYGPTAGAA
LASHMDVDKVAFTGSTDTGKIILELAAKSNLKTVTLELGGKSPFIIMGDADVDHAVELAHFALFFNQGQC
CCAGSRTFVHERVYDEFVEKSKARALKRVVGDPFRKGVEQGPQIDDEQFNKILRYIRSGVDSGANLVTGG
DRLGDKGYYIQPTIFSDVQDGMKIAQEEIFGPVQSILKFKDLNEVIKRANASQYGLAAGVFTNNLDTANT
LARALRVGTVWTNCFDIFDAAIPFGGYKMSGHGREKGIDSLKNYLQVKAVVTPIKNAAWL
